# Synthesis of some novel coumarin isoxazol sulfonamide hybrid compounds, 3D-QSAR studies, and antibacterial evaluation

**DOI:** 10.1038/s41598-021-99618-w

**Published:** 2021-10-11

**Authors:** Sheida Nasr Esfahani, Mohammad Sadegh Damavandi, Parisa Sadeghi, Zahrasadat Nazifi, Azhar Salari-Jazi, Ahmad Reza Massah

**Affiliations:** 1 Department of Chemistry, Shahreza Branch, Islamic Azad University, 86145-311 Isfahan, Iran; 2 Department of Microbiology, School of Medicine, Isfahan University of Medical Sciences, Isfahan, Iran; 3 Department of Drug Development and Innovation, Behban Pharmed Lotus, Tehran, Iran; 4 Razi Chemistry Research Center, Shahreza Branch, Islamic Azad University, Isfahan, Iran

**Keywords:** Biochemistry, Computational biology and bioinformatics, Antimicrobials, Medicinal chemistry

## Abstract

With the progressive and ever-increasing antibacterial resistance pathway, the need for novel antibiotic design becomes critical. Sulfonamides are one of the more effective antibiotics against bacteria. In this work, several novel sulfonamide hybrids including coumarin and isoxazole group were synthesized in five steps starting from coumarin-3-carboxylic acid and 3-amino-5-methyl isoxazole and assayed for antibacterial activity. The samples were obtained in good to high yield and characterized by FT-IR, ^13^C-NMR, ^1^H-NMR, CHN and melting point techniques. 3D-QSAR is a fast, easy, cost-effective, and high throughput screening method to predict the effect of the compound's efficacy, which notably decreases the needed price for experimental drug assay. The 3D-QSAR model displayed acceptable predictive and descriptive capability to find r2 and q2 the pMIC of the designed compound. Key descriptors, which robustly depend on antibacterial activity, perhaps were explained by this method. According to this model, among the synthesized sulfonamide hybrids, **9b** and **9f** had the highest effect on the gram-negative and gram-positive bacteria based on the pMIC. The 3D-QSAR results were confirmed in the experimental assays, demonstrating that our model is useful for developing new antibacterial agents. The work proposes a computationally-driven strategy for designing and discovering new sulfonamide scaffold for bacterial inhibition.

## Introduction

The antibacterial resistances which were exacerbated by the use and abuse of antibiotics, have become a significant primary public concern. Every day, there are some bacterial reports that are resistant to antimicrobial agents and many times these bacteria are MDR or XDR and PDR, therefore antibacterial drugs have been losing their efficacy against infections^1^.

Sulfonamides are Dihydropteroate synthase inhibitors that have been broadly performed to treat bacterial infections. These agents contend with aminobenzoic acid and consequently block the nucleic acids and proteins syntheses, so inhibit the microorganism's grows^2^. Sulfonamides have drawn far too much attention due to their other medicinal application such as anticonvulsants^3^, protease inhibitors^4^, anti-inflammatory^5^, antitumor^6^ and anticancer agents^7^. These reasons have prompted numerous attempts to design and synthesize new structural sulfonamides derivatives with exceptional, extended-spectrum and low toxic activity.

Coumarins with synthetic and natural origin constitute a large group of heterocyclic compounds. Many compounds derived from coumarin show varied biological and pharmaceutical activities^8^. Coumarin derivatives have been used as antibacterial^9–12^, anticoagulants^13^, antioxidant^14^ and antifungal^15^.

Isoxazoles, are an important group of heterocyclic compounds that have interesting biological activities^16^. Several compounds derived from isoxazole exhibited biological activity such as antibacterial^17,18^, anticancer^19^, anti-inflammatory^20^, antipsychotic^21^, pesticides^22^ and anti-rheumatic^23^.

**Figure 1:**
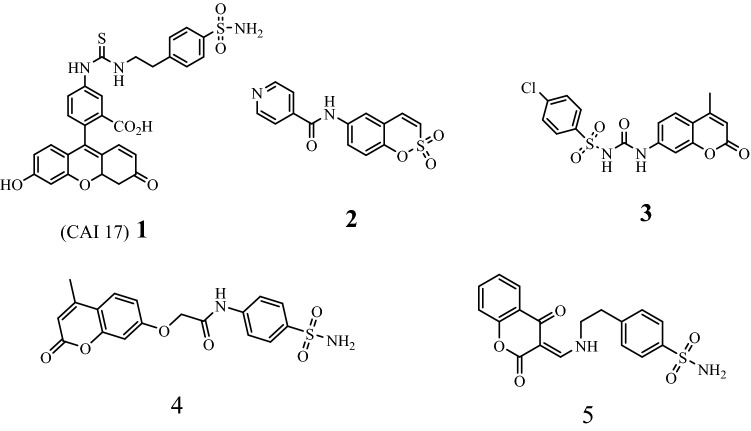
Structure of biologically active coumarin-sulfonamide hybrids.

Combining two or more pharmacological groups into a single molecule is an emerging drug discovery strategy and medicinal chemistry^24^. Coumarin sulfonamide hybrids are excellent compounds which have several applications in pharmacology^25–27^. It was found that CAI17 (**1**), as a coumarin sulfonamide hybrid structure, showed anti-metastatic activity. Sulfocoumarin (**2**) and coumarin sulfonamide (**3**) showed significant selectivity versus carbonic anhydrase IX and XII^28^. Also, the hybrid structures **4** and **5** exhibited antioxidant and antibacterial activities^29,30^, respectively (Fig. 1).

**Figure 2:**
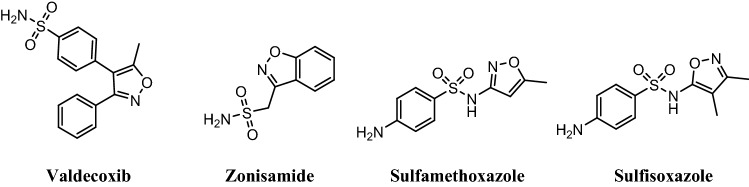
Structure of biologically active sulfonamide-isoxazole hybrids.

Due to the biological activity of isoxazoles and sulfonamides, several sulfonamides bearing isoxazole moiety have been designed and synthesized as pharmacological active compounds. Valdecoxib, as an isoxazole sulfonamide hybrid drug, demonstrated as a selective COX-2 inhibitor^31^. Zonisamide was showed anticonvulsant and antiobesity properties^32,33^. Sulfamethoxazole and sulfisoxazole were known as antibacterial isoxazole sulfonamide hybrid drugs^34^ (Fig. 2).

**Figure 3:**
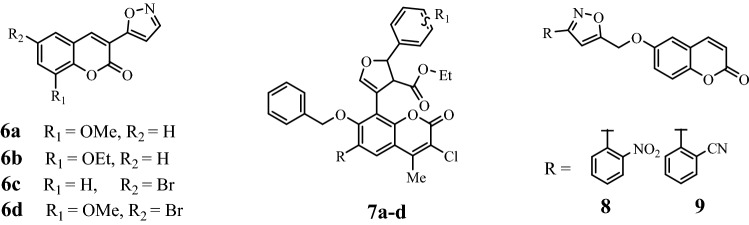
Structure of biologically active coumarin-isoxazole hybrids.

Isoxazole coumarin is an important hybrid structure that is an essential and integral part of various therapeutic scaffolds. Recently, Khaled et al*.*, was studied the antioxidant activity of several coumarin isoxazole derivatives using DPPH as radical scavenging^35^. They found that the target compounds (**6a–d**) have broad range of activities in comparison with silymarin which among them the methoxy derivative (**6d**) was the most potent one. Also, several antibacterial coumarin-based isoxazoles (**7a–d**) were synthesized by Suresh et al*.*^36^. In another work, isoxazoles connected 6-hydroxycoumarin were synthesized and studied their cytotoxic activity^37^. The best and selective activity was observed for compound **8** and **9** against prostate (PC-3) cancer cell line (Fig. 3).

Novel drug introducing process to market entail significant time and money investment. A drug's development and introduction into the market are estimated to spend about 2.6 billion dollars and the price has risen by almost 150 percent in the last ten years. However, the failure rate has risen to almost 90%^38^. There are a divers of computer-aided drug discovery (CADD) methods that can be used to create bioactive molecules. Based on the molecular interaction nature, Three-dimensional quantitative structure–activity relationship (3D-QSAR) analyses. This calculation offers plenty of knowledge about the precise molecular features that prerequisite for biological activity and can be used as a major predictive method for pharmaceutical design. The relationship between the configuration of the ligand and its biological activity was quantified by using QSAR^39^.

In continuance of our works on the synthesis and computational studies of small biologically active compounds^40–45^, 3D-QSAR models were performed to predict novel coumarin Isoxazol sulfonamide hybrids possible MIC on the bacteria. Also, the antibacterial activity of the designed and synthesized sulfonamides hybrids was assessed. The predicted MIC and experimental MIC were compared and the accuracy of 3D-QSAR was discussed.

## Results and discussion

### Bioactive conformation hunt, compound alignment

**Figure 4:**
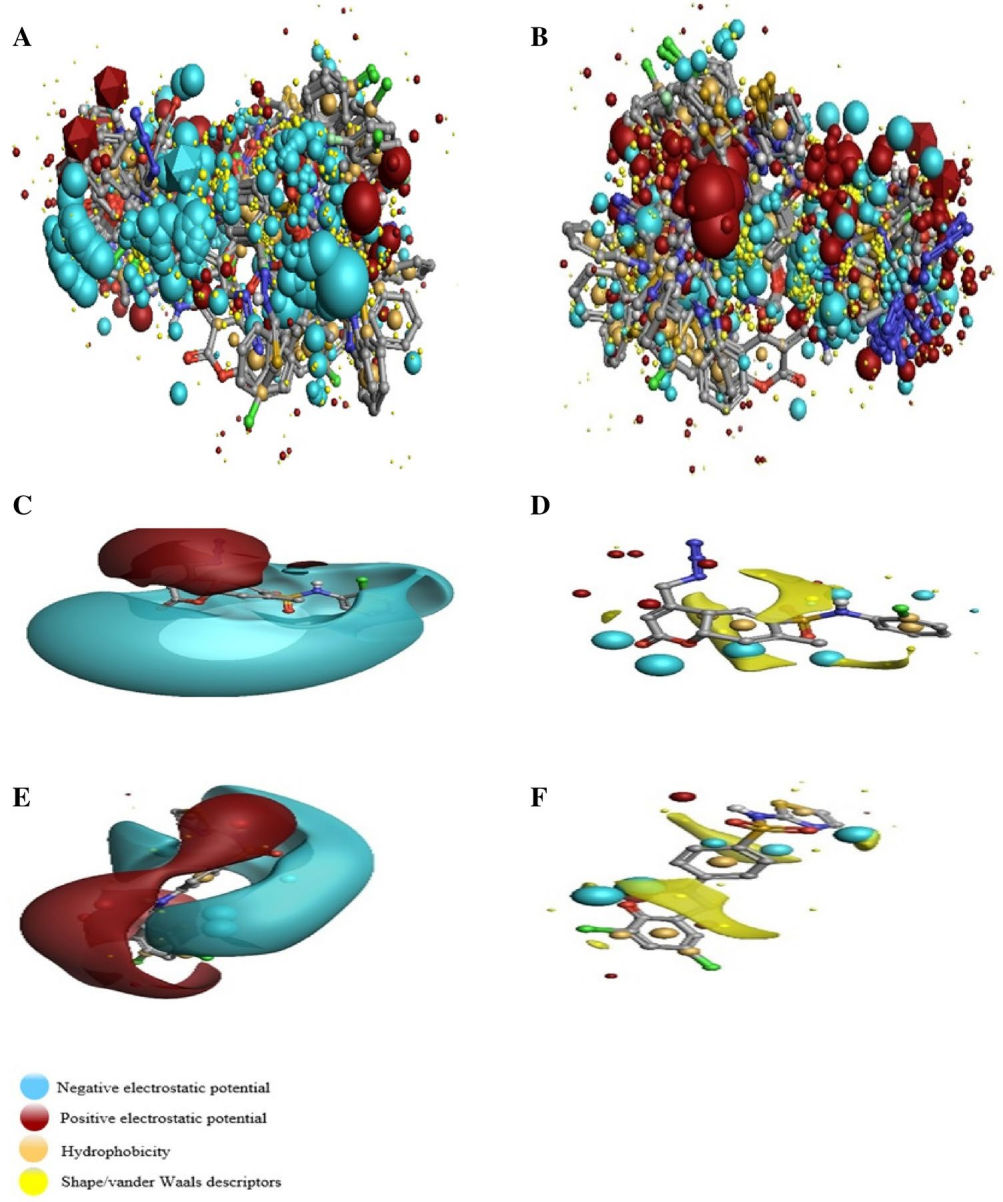
(**A**, **B**) The aligned training set compounds’ Molecular exhibition with regard to their field points. Molecular representation of high and low active training set compounds with respective biological activity (pMIC). (**C–F**) The molecular regions interacting with positive or H-bond donors of the target were to be presented by cyan color, which show negative field points. Positive field points were to be exhibited by red color that indicates possible molecular regions interacting with negative or H-bond acceptor of the target protein. Gold color indicates hydrophobic field points, which shows high polarizability or hydrophobicity areas, and yellow color presented Van der Waal field points. (**C**) and (**D**) belong to the *S. aureus* 3D-QSAR, (**E**) and (**F**) belong to the *E. coli* 3D-QSAR*.*

Dihydropteroate Synthase inhibitors’ affinities were predicted by using 3D-QSAR model. The 3D field points pattern for protein was discovered according to the established proposition of bioactive conformations that were annotated by their measured field points. This 3-Dimensional field point pattern assisted to determine compounds similarity and compounds common pharmacophore for discovering their pMIC. The aligned compounds with regard to their molecular field points are in the Fig. 4. All aligned compounds with or without field points is in the Figure S1.

### 3D QSAR model development and statistical analysis

**Figure 5:**
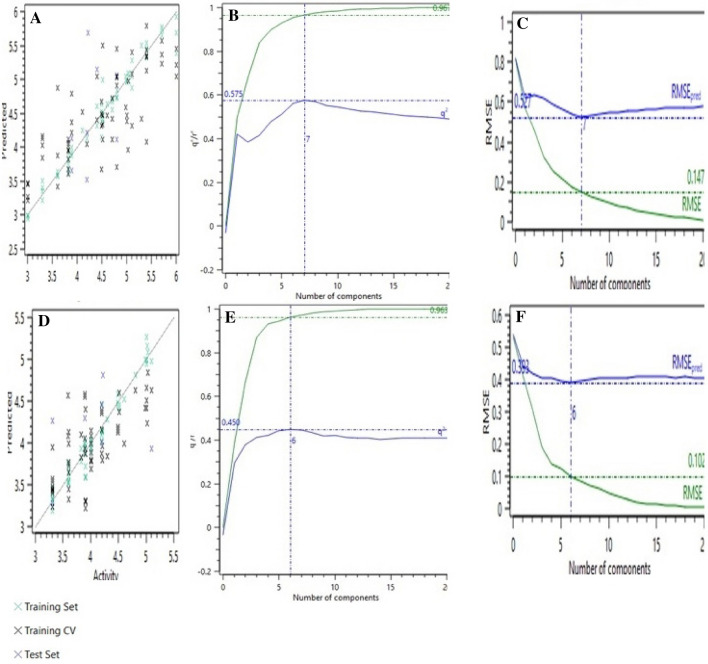
The left column shows an activity interactive graph plot among experimental and predicted activity. The Middle column presented 3D-qsar model performance graph plot of *r*^2^ and *q*^2^ and the number of components. The right column shows the RMSE. (**A**–**F**) Belongs to *S. aureus*, and *E. coli*, respectively.

To build 3D-QSAR model, the Field points based chemical descriptors on all aligned compounds were performed. The 3D-QSAR model components had the strong predictive and informative ability that presented a good regression coefficient *r*^2^, cross-validation regression coefficient *q*^2^ and root mean square error (RMSE) values for the training and the cross-validated training set (in both *E. coli* and *S. aureus*). Similar results in the test set obtained, which firmly verify the 3D-QSAR model for the prediction set. The compared predictive versus experimental plot shown in Fig. 5.

### SAR mechanism of field points

The QSAR model was developed in three dimensions to better explain the SAR mechanism of sulfonamides analogs. The activity-related field points that are coefficient for all compounds and training set were explored to discover the mentioned SAR mechanism. This model described the areas where local fields had a significant impact on biological activity. The larger area points presented a deeper correspondence between electrostatic/steric fields, resulting in greater affinity values and bioactivity. The QSAR model points were superposed to the reference compound's configuration to realize the space field point's localization. The high coefficient and variance field was caused by the notable correlating criteria in the intensity model. In a robust model, the large coefficient points were emitted by significant correlating parameters. The structural analysis showed the proven QSAR model was dominated by the big steric + scale, therefore stated more steric produces higher activity. The electrostatic coefficient also serves a part in the activity effects of substituents. Moreover, the electrostatic coefficient also plays a role in substituent activity effects. The high variance (electrostatic & steric) field points denote the area with a lot of changes, while low variance field points reflect a domain with less or no changes (Figure S42).

### SAR mechanism identification through activity-Atlas visualization

The Dihydropteroate Synthase principal features affinity for its ligands that inhibit this enzyme behavior were discovered by SAR analysis and were visualized by activity atlas. The activity atlas visualization method is a qualitative and beneficial procedure to summarize SAR data into 3D map. Activity atlas displays the prediction set's resemblance to field points, average electrostatic, average activity, and other SAR properties high active compounds.

### Average of actives analysis results

**Figure 6:**
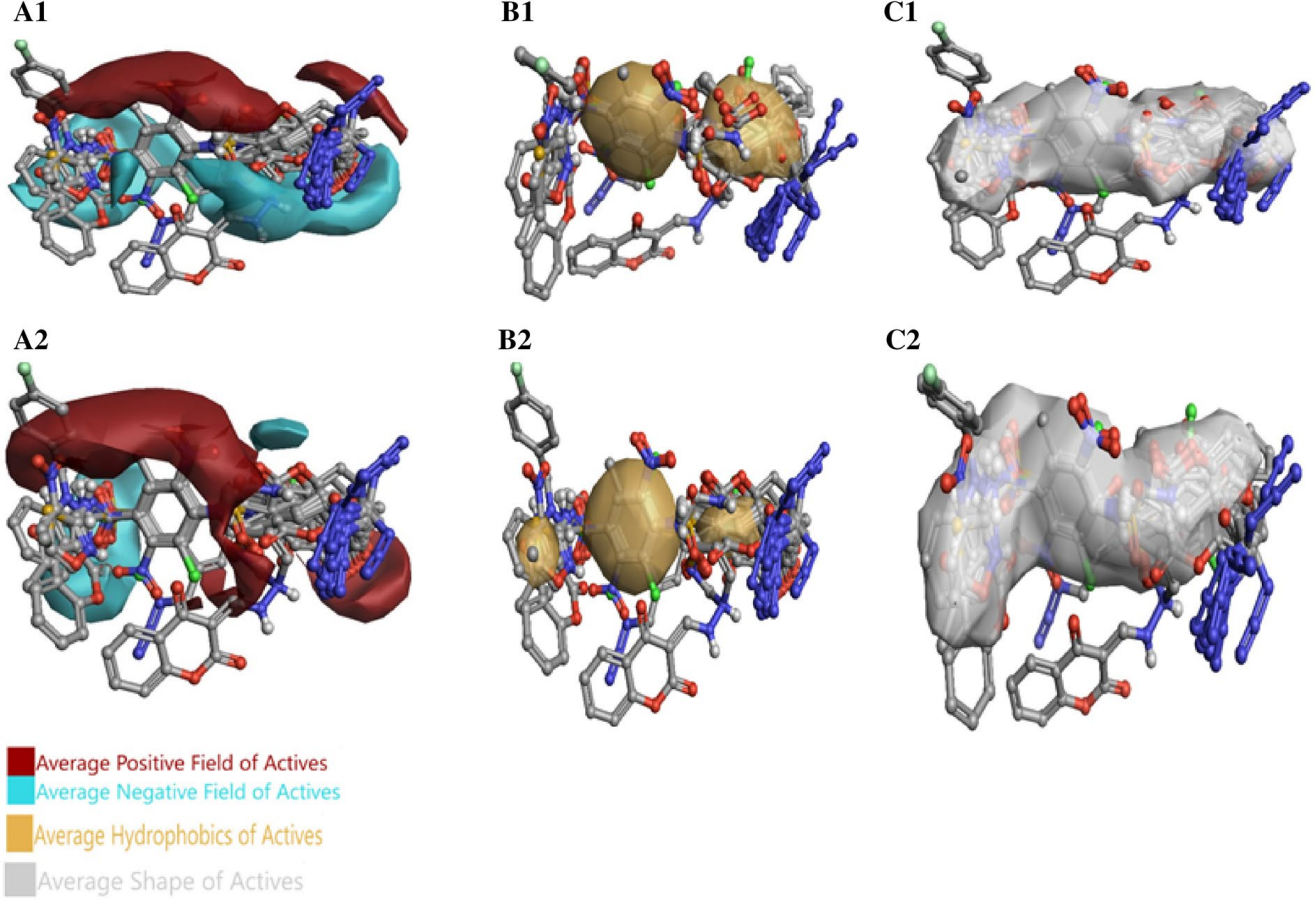
Molecular insight of SAR mechanism models demonstrate an average of actives analysis. (**A**) Active molecules generally have a positive field in red color area, and a negative field in the cyan color region, (**B**) active molecules commonly make hydrophobic interaction in the yellow color area, (**C**) average shape of active molecules. The A1–C1 belongs to the *S. aureus* and *E.* coli results placed in the A2–C2.

The identified fields along with shape and hydrophobic interactions depend on the high biological activity, and it stated new molecules which show negative or positive fields in the same or similar area that must be supposed as an active molecule Fig. 6.

### Validation of the 3D-QSAR

Predicted versus experimental activity plots of the training and test set's molecules distinctly placed in the (Figure S43). Both two models of the *S. aureus* and *E. coli* displayed that observed and estimated activity were highly correlated in the training and test set's compounds. Also, the compounds’ predicted activity was remarkably approach to the experimental activity. The *r*^2^ of training and test sets of all models were beyond 0.6 so all of these data verify that this model is the accurate and applicable to predict MIC of the compounds. All of these observations indicate that the model is capable of accurately predicting the pMIC of compounds.

### The compounds predicted activity

All compounds predicted activity had an excellent result, especially about the which was/or were more effective than the other compounds Table 1.

**Table 1:** Predicted of biological activity to prediction set.

Compound no	Predicted pMIC (pMIC = −log (MIC))	
	pMIC *S. aureus*	pMIC *E. coli*
**9a**	4.1	4.1
**9b**	4.2	4.3
**9c**	3.9	4.1
**9d**	4.2	4.2
**9e**	3.9	4.3
**9f**	4.3	4.4
**9g**	3.7	4.2
**9h**	4.2	4.2
**9i**	4.2	4.1
**9j**	4.1	4.2
**9k**	4.1	3.9
**9l**	3.9	3.6

### Chemistry

**Figure 7:**
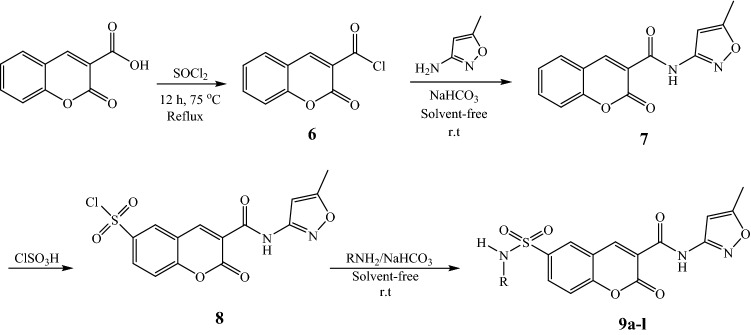
Synthetic route of the synthesis of coumarin sulfonamide isoxazol hybrids **9a**–**l**.

The general pathway for the synthesis of Isoxazol sulfamoyl chromene carboxamides **4a–l** was shown in Fig. 7.

To begin, cumarin-3-carboxylic acid chloride was prepared by the reaction of cumarin-3-carboxylic acid with SOCl_2_. In the second step, the obtained cumarin acid chloride was reacted with 3-amino-5-methyl isoxazole to produce the corresponding amide **7** in high yield and purity. In this step, using sodium hydrogen carbonate as base, solvent-free conditions and room temperature are showing that the reaction was carried out under green conditions. Then, chlorosulfonic acid was used for the chlorosulfonating of the obtained amide (**7**). The corresponding sulfonylchloride (**8**) was very pure and used directly in next step. Finally, the obtained Isoxazol coumarin sulfonylchloride (**8**) was combined with different amines to form the corresponding sulfonamides (**9a–l**). The reaction was carried out in the presence of a sodium hydrogen carbonate as a green base at ambient temperature in the absence of any solvent. All of the coumarin sulfonamide isoxazol hybrid compounds (**9a–l**) were obtained in high purity after an easy work-up, just by adding water and simple filtration. It means that this step as the same as the second step is completely in agreement with green chemistry. Several aromatic and aliphatic amines were tested to extend the synthetic utility of the procedure (Table 2). As the results is shown, aromatic amines containing electron-donating and electron-withdrawing substituent and also aliphatic amines are reacted with sulfonyl chloride **3** in 10–30 min and the corresponding sulfonamides were obtained in 85–95% yield with great purity (Figure S1–S41). In comparison, anilines with electron-donating groups such as methoxy and methyl reacted with sulfonyl chloride faster than those with the electron-withdrawing group such as Cl and Br and the products were obtained in higher yields.

**Table 2:**
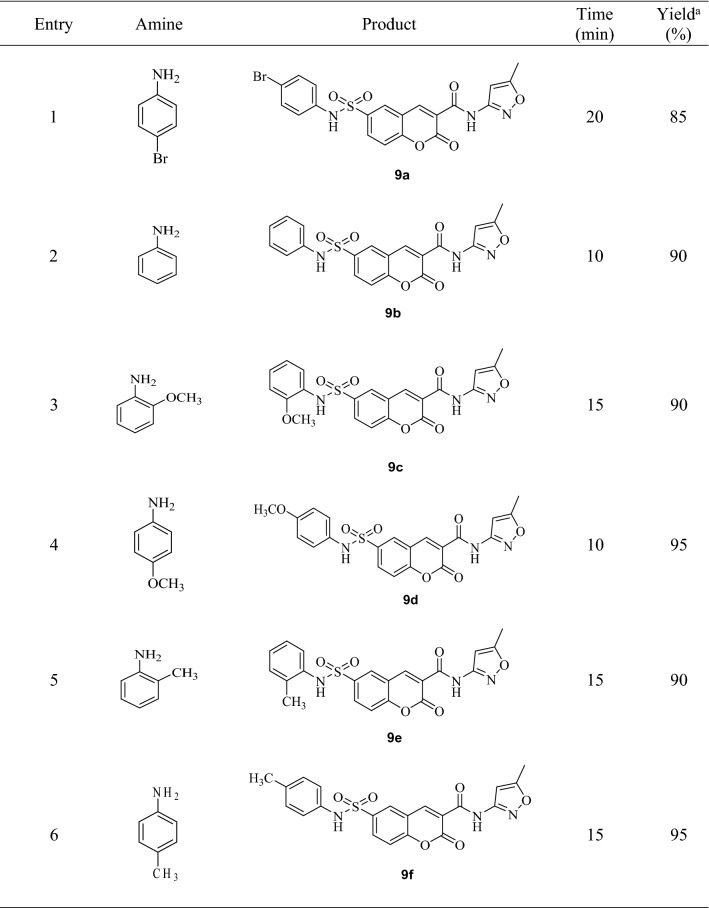

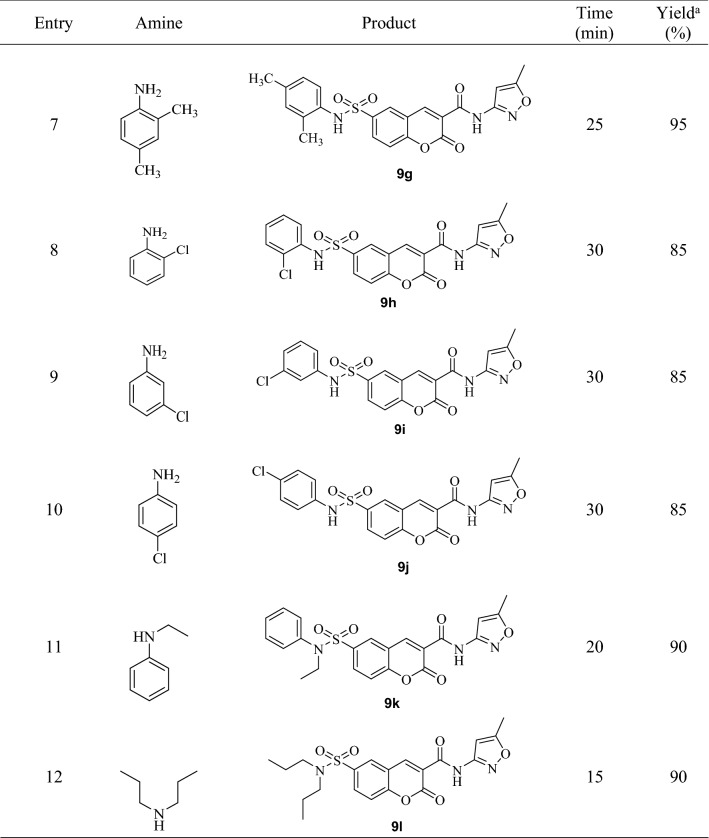
Synthesis of coumarin sulfonamide isoxazol hybrids 9**a**–**l**.

^a^Isolated yield.

### Biological evaluation

The antibacterial activity of the synthesized sulfonamide base hybrids (**9a–l**) was investigated against *E. coli* and *S. aureus* by applying the agar diffusion method. As the results was shown, aromatic amines with no substituent (entry **2**) and methyl substituent at the 4-position of phenyl ring (entry **6**) were the most potent compounds against both *E.coli* and *S. aureus* strains (Table 3). Derivatives including halogen groups (Cl, Br) in *para* position (entry **1**, **10**), showed higher antibacterial activity against *S. aureus* than *E coli*. This indicated that compounds with electron-withdrawing or electron-donating groups at 4-position showed better inhibitory activities than those compounds with no substituent. Compounds with chlorin atom at *orto* position of phenyl ring (entry **8**), could efficiently improve the antibacterial activity against *E. coli* but this substituent is not suitable against *S. aureus.* The introduction of methoxy group at the *orto* or *meta* positions of phenyl ring (entry **3**, **4**), led to a reduction in activity. Also, the replacement of aromatic amines with aliphatic amine (entry **12**) improved the inhibitory activity against *E. coli*, while against *S. aureus* is not tolerable.

**Table 3:** Antimicrobial activities of the coumarin 3-carbamide sulfonamides against the pathological organisms based on agar diffusion method.

Compound no	Zone of inhibition^a^									
	Gram positive‏					Gram negative				
	*S. aureus*					*E. coli*				
	5 mg/ml	2.5 mg/ml	1.25 mg/ml	0.62 mg/ml	0.31 mg/ml	5 mg/ml	2.5 mg/ml	1.25 mg/ml	0.62 mg/ml	0.31 mg/ml
**9a**	28	28	16	–	–	22	14	10	–	–
**9b**	29	28	28	19	–	22	18	16	15	–
**9c**	15	15	–	–	–	10	–	–	–	–
**9d**	10	–	–	–	–	11	–	–	–	–
**9e**	12	11	10	–	–	20	14	10	–	–
**9f**	28	28	26	22	20	23	22	22	20	–
**9g**	16	15	15	–	–	17	15	–	–	–
**9h**	11	–	–	–	–	22	20	17	15	–
**9i**	28	28	27	–	–	25	24	23	–	–
**9j**	19	17	16	13	–	22	20	19	–	–
**9k**	13	11	–	–	–	20	13	10	–	–
**9l**	14	–	–	–	–	22	18	17	15	–

^a^Mean zone of inhibition in mm beyond well diameter (6 mm) produced on a range of clinically pathogenic microorganisms.

#### MIC

MIC of All the new synthesized compounds was assessed by microdilution broth. MIC results presented that most compounds are moderately active against bacteria except **9b** and **9f** that have the robust antibacterial effect on the *E. coli* and *S. aureus*. **9f** had a consequential effect on the *E. coli* (Table 4).

**Table 4:** Minimum inhibitory concentrations (MIC) in µg/ml of compounds tested in microdilution method.

Compound no	*E. coli*	*S. aureus*
**9a**	128	256
**9b**	64	128
**9c**	128	> 512
**9d**	128	256
**9e**	128	> 512
**9f**	32	128
**9g**	128	128
**9h**	128	> 512
**9i**	> 512	128
**9j**	256	128
**9k**	256	256
**9l**	> 512	128

## Conclusion

The researched work focused on the field-based 3D QSAR model expansion on a series of coumarin isoxazol sulfonamide hybrids to investigate the mechanism of sulfonamide inhibition on the bacteria development of the new sulfonamides with enhanced power of the antibacterial effect. The elucidated action mechanism clarified the SAR and, thus, may be accelerate design and improve the structure of novel and potent sulfonamide ligand against bacteria. The structure study and chemical field analyses made it bright to assess which structure of sulfonamides block bacterial growth. The obtained outcomes also suggest potential insight for the area where the active molecules lie and also implicate the average active molecules shape. The positive and negative charges, such as hydrophobic regions, were demonstrated. This utilized method would assist to consider the model predicted compounds and potential modifications assumption. These variables make fitted model to compounds and predict required changes to raise their bioactivity.

The good *r*^2^, *q*^2^ and RMSE values for the training and the CV training set (in both E. coli and S. aureus) gained. Accordingly, some coumarin 3-carbamide sulfonamides derived from 3-amino-5-methyl isoxazole were synthesized and evaluated for the antibacterial activity. The obtained products were characterized using melting point, ^13^C NMR, ^1^H NMR and FT-IR techniques. All the compounds were synthesized very pure with an easy procedure. Also, the in vitro antibacterial activities of the synthesized sulfonamides have been studied on the *S. aureus* and *E. coli* strains. The predicted activity of the compounds was near to the MIC activity and in accordance with the expectation (based on the predicted activity of bioactivity) **9b** and **9f** compounds had better antibacterial activity than the others, which made them potent inhibitors against bacteria. It seems like methyl substituent at the 4 positions of anilines (**9f**) and also derivatives without substituent (**9b**), create the most potency against both gram-negative and positive bacteria. Predicted activity in 3D-QSAR model successfully predicted the arrangement of the efficacy of the synthesized sulfonamide special about the high influence of the **9f** and **9b** compounds on the gram-negative and gram-positive bacteria. These two compounds had the more predicted activity than the other. On the other hand, our 3D-QSAR capability to predict the bioactivity of sulfonamides not only in the computational model was excellent but also in the in vitro experimental test had acceptable outcomes.

## Methods

### In silico assay

#### QSAR, 3D-QSAR data set

Data set was gathered against Dihydropteroate Synthase (CHEMBL4032, CHEMBL1075045) for sulfonamides from ChEMBL 20 database and articles^30,46–52^. The database includes compounds that had the antibacterial effect and sulfonamides functional group. The initial dataset derived from MIC was selected for further investigation on the dataset. Compounds which had not the MIC were omitted^53^. Then 65, and 72 Compounds were gained for *S. aureus* and *E. coli*, respectively. All collected data set placed in the supplementary 2 file.

### 3D-QSAR; conformation hunt, pharmacophore generation, alignment, and built model calculations

The accumulated CSV format was transformed to SDF format. All articles extracted compounds were created in the Marvin Sketch program. Discovery Studio program^54^ applied to minimize (CHARMm force field) and add hydrogen (in pH = 7) to the Marvin Sketch created compound. All collected compounds (compounds from ChEMBL database and articles) were gathered in the one SDF format dataset. The crystallize targeted protein (PDB = 3TYE) was extracted from the PDB site and separated into protein receptor and ligand references. The bound drug was utilized as a reference ligand to produce field pharmacophores and subsequently as a bioactive reference conformation, while the target receptor was used as a protein receptor excluding volume. The reference conformation was also annotated with its computed field points, which were generated in 3D field point pattern. Filed pharmacophore, bioactive reference conformation, target receptor and references drug were employed to generate GRID. XED (eXtended Electron Distribution) force field was applied to make field points. As a result, three divers molecular fields including ‘Shape’ (van der Waals), positive and negative electrostatic, and ‘hydrophobic’ fields (a density function associated with steric bulk and hydrophobicity) were computed. The filed points template provides a standardized description of the electrostatics, compound’s shape, and hydrophobicity. Following that, the reference conformer was utilized to align the compounds in the training and test sets by using Maximum Common Substructure (MCS) and customized thresholds. The conformation hunt was carried out by using very accurate and slow calculation procedure with a maximum number of conformations produced for every molecule set to 1000. To create the 3D-QSAR model, the best fitting low energy conformations for the template were used. The compounds data set's experimental behavior was converted to positive logarithmic scale. *E. coli* and *S. aureus* pMIC, which is produced via formula of pMIC = −log (MIC), was used as an associate variable. In a ratio of 80 percent and 20%, total molecules were divided into the training and test sets. gradient conformer minimization and RMSD Cutoffs were established to 0.1 kcal/mol and 0.5 Å for atomic positions, respectively.

The Forge utilized a 50% Field similarity plus 50% Dice volume similarity. The partial least squares (PLS) regression method was utilized to construct the model using Forge's field QSAR module, particularly the SIMPLS algorithm^55,56^.

### Validation of the 3D-QSAR

The correlation coefficient (*r*^2^), the cross-validation regression coefficient *q*^2^ and the root mean square error (RMSE) were considered to the 3D-QSAR model predictive potential^54^.

### Visualization of SAR activity Atlas models

To qualitatively visualize the training dataset, the Bayesian technique was used. Bayesian metod offered comprehensive interpretation of the electrostatic, hydrophobic and shape characteristics, and constitute selected compounds ’SAR. The three-dimensional analysis of these models will provide valuable detail. The activity atlas study offered a range of interrelated biochemical outcomes which comprising activity regions explore analyses and an average shape of active. The average of actives revealed the active compounds’ common part. regions explored investigation demonstrated the areas of the aligned compounds which have been thoroughly studied^57^.

### Experimental

All commercially chemicals were prepared from the Merck Chemical Company. The structure of synthesized compounds was confirmed using FT-IR spectroscopy (Nicolet 400D, KBr matrix, 4000–400 cm^−1^), melting points obtained with an Amstead Electro Thermal 9200 apparatus, and nuclear magnetic resonance (^1^H NMR, ^13^C NMR) spectra (Bruker DRX-400 Avance spectrometer at 400 and 100 MHz, respectively. The purity of products and also reaction progress evaluation were determined using thin layer chromatography on silica-gel Polygram SILG/UV 254 plates (Merck).

#### Synthesis of 2-oxo-2H-chromene-3-carbonyl chloride (1)

A mixture of SOCl_2_ (4.8 mmol) and 2-oxo-2H-chromene-3-carboxylic acid (4 mmol) was stirred for 12 h at 75 ºC. After extra SOCl_2_distillation , high purity light yellow solid product was obtained; M.p = 158–160 °C (M.p_Lit_ = 158–161 °C)^45^.

#### Synthesis of *N*-(5-methylisoxazol-3-yl)-2-oxo-2H-chromene-3-carboxamide (2)

A mixture of synthesized chromene-3-carbonyl chloride (3 mmol), sodium hydrogen carbonate (NaHCO_3_, 3 mmol) and 3-amino-5-methyl isoxazole (3 mmol) was triturated at room temperature under solvent-free conditions. After completion of the reaction as it was monitored by TLC, 25 mL distilled water was added, stirred for 15 min, and filtrated. The very pure generated product directly employed for the next step.

Light Cream; M.p = 247–250 °C (M.p_Lit_ = 230 °C)^46^; R_f_ = 0.28 (EA: *n*-Hex; 30:70); IR (KBr, cm^−1^) = 3228 (N–H), 1713, 1677 (C=O), 1611, 1563, 761.

#### Synthesis of 3-((5-methylisoxazol-3-yl) carbamoyl)-2-oxo-2H-chromene-6-sulfonyl chloride (3)

Chlorosulfonic acid (20 mmol) was slowly added and stirred to compound **2** (2 mmol) at 0 ºC and for 0.5 h. The stirred process was continued for 1 h at room temperature and 8 h at 60 ºC. Then, the mixture was added drop wise to ice bath, stirred for 5 min, filtered and washed with water until neutralized. The product was obtained very pure and characterized by FT-IR, melting point, ^13^C NMR, and ^1^H NMR methods.

Dark Cream; M.p = 216–219 °C; R_f_ = 0.44 (EA: *n*-Hex; 30:70); IR (KBr, cm^−1^) = 3266 (N–H), 1731, 1680 (C=O), 1612, 1543, 1379, 1173 (SO_2_); ^1^H NMR (400 MHz, DMSO-d_6_) *δ* (ppm) = 2.43 (s, 3H_CH3_), 6.78 (s, 1H_Ar_), 7.50 (d, 1H_Ar_, *J* = 8.8 Hz), 7.96 (dd, 1H_Ar_, *J* = 2.0), 8.24 (d, 1H_Ar_, *J* = 1.6 Hz), 9.00 (d, 1H_Ar_, *J* = 2.0 Hz), 11.17 (s, 1H_N–H_), 14.34 (s, 1H_N–H_); ^13^C NMR (100 MHz, DMSO-d_6_) *δ *(ppm) = 12.6, 96.7, 116.6, 118.0, 119.5, 127.6, 132.4, 145.0, 148.8, 154.4, 157.6, 160.1, 160.8, 170.9.

#### General process for the Isoxazol sulfamoyl chromene carboxamide synthesis (4a–l)

A mixture of 3-((5-methylisoxazol-3-yl) carbamoyl)-2-oxo-2H-chromene-6-sulfonyl chloride (**3**) (1 mmol), NaHCO_3_ (1 mmol) and amine (1 mmol) was grinded in a mortar at room temperature. After completion of the reaction (monitored by TLC), the distilled water was added (25 mL), stirred for 5 min, filtered and washed with enough water until neutralized. The final product was dried and characterized by melting point, FT-IR, NMR and CHN analysis methods.

### Spectral data of the synthesized products

#### 6-(*N*-(4-bromophenyl)sulfamoyl)-*N*-(5-methylisoxazol-3-yl)-2-oxo-2H-chromene-3-carboxamide (4a)

Light Yellow Solid; M.p = 236–240 °C; R_f_ = 0.24 (EA: *n*-Hex; 35:65); IR (KBr, cm^−1^) = 3275 (N–H), 1731, 1680 (C=O), 1334, 1157 (SO_2_); ^1^H NMR (400 MHz, DMSO-d_6_) *δ* (ppm) = 2.45 (s, 3H_CH3_), 6.79 (s, 1H_Ar_), 7.07 (d, 2H_Ar_, *J* = 9.6 Hz), 7.43 (d, 2H_Ar_, *J* = 9.6 Hz), 7.72 (d, 1H_Ar_, *J* = 8.8 Hz), 8.04 (dd, 1H_Ar_, *J* = 2.0 Hz), 8.50 (d, 1H_Ar_, *J* = 2.4 Hz), 9.00 (s, 1H_Ar_), 10.70 (s, 1H_N–H_), 11.11 (s, 1H_N–H_); ^13^C NMR (100 MHz, DMSO-d_6_) *δ* (ppm) = 12.6, 96.8, 117.0, 118.4, 118.9, 121.5, 122.5, 130.0, 132.0, 132.6, 136.2, 137.1, 147.3, 156.6, 157.7, 159.9, 160.1, 170.9. Anal. Calcd. for C_16_H_12_BrN_3_O_6_S, %: C, 47.63; H, 2.80; N, 8.33. Found, %: C, 47.51; H, 2.96; N, 8.47.

#### 6-(*N*-(phenyl)sulfamoyl)-*N*-(5-methylisoxazol-3-yl)-2-oxo-2H-chromene-3carboxamide (4b)

Cream Solid; M.p = 250–253 °C; R_f_ = 0.2 (EA: *n*-Hex; 35:65) IR (KBr, cm^−1^) = 3249 (N–H), 1739, 1691 (C=O), 1337, 1151 (SO_2_); ^1^H NMR (400 MHz, DMSO-d_6_) *δ* (ppm) = 2.44 (s, 3H_CH3_), 6.78 (s, 1H_Ar_), 7.04 (t, 1H_Ar_), 7.12 (d, 2H_Ar_, *J* = 7.6 Hz), 7.24 (t, 2H_Ar_), 7.70 (d, 1H_Ar_, *J* = 8.8 Hz), 8.04 (dd, 1H_Ar_, *J*_1_ = *J*_2_ = 2.0 Hz), 8.49 (d, 1H_Ar_, *J* = 2.4 Hz), 8.99 (s, 1H_Ar_), 10.51 (s, 1H_N–H_), 11.10 (s, 1H_N–H_); ^13^C NMR (100 MHz, DMSO-d_6_) *δ* (ppm) = 12.6, 96.8, 118.3, 118.9, 120.7, 121.5, 124.9, 129.7, 129.9, 132.1, 136.6, 137.7, 147.2, 156.5, 157.7, 159.8, 160.1, 170.9. Anal. Calcd. for C_20_H_15_N_3_O_6_S, %: C, 56.47; H, 3.55; N, 9.88. Found, %: C, 56.52; H, 3.39; N, 3.43.

#### 6-(2-Methoxy-phenylsulfamoyl)-2-oxo-2H-chromene-3-carboxylic acid (5-methyl-isoxazol-3-yl)-amide (4c)

Dark Cream Solid; M.p = 206–210 °C; R_f_ = 0.12 (EA: *n*-Hex; 35:65); IR (KBr, cm^−1^) = 3260 (N–H), 1726, 1696 (C=O), 1341, 1159 (SO_2_); ^1^H NMR (400 MHz, DMSO-d_6_) *δ* (ppm) = 2.45 (s, 3H_CH3_), 3.46 (s, 3H_OCH3_), 6.79 (s, 1H_Ar_), 6.88 (m, 2H_Ar_), 7.14 (m, 1H_Ar_), 7.24 (m, 1H_Ar_), 7.71 (d, 1H_Ar_, *J* = 8.8 Hz), 8.03 (dd, 1H_Ar_, *J*_1_ = *J*_2_ = 2.0 Hz), 8.37 (d, 1H_Ar_, *J* = 2.0 Hz), 8.97 (d, 1H_Ar_, *J* = 2.8 Hz), 9.77 (s, 1H_N–H_), 11.13 (s, 1H_N–H_); ^13^C NMR (100 MHz, DMSO-d_6_) *δ* (ppm) = 12.6, 55.8, 96.8, 112.3, 117.7, 118.5, 120.9, 121.3, 125.1, 126.7, 127.8, 129.6, 132.5, 137.9, 147.3, 153.2, 156.3, 157.7, 160.0, 160.2, 170.9. Anal. Calcd. for C_21_H_17_N_3_O_7_S, %: C, 55.38; H, 3.76; N, 9.23. Found, %: C, 55.48; H, 3.60; N, 9.38.

#### 6-(4-Methoxy-phenylsulfamoyl)-2-oxo-2H-chromene-3-carboxylic acid (5-methyl-isoxazol-3-yl)-amide (4d)

Light Green Solid; M.p = 244–248 °C; R_f_ = 0.12 (EA: *n*-Hex; 35:65); IR (KBr, cm^−1^) = 3216 (N–H), 1717, 1672 (C=O), 1340, 1156 (SO_2_); ^1^H NMR (400 MHz, DMSO-d_6_) *δ* (ppm) = 2.44 (s, 3H_CH3_), 3.67 (s, 3H_OCH3_), 6.79 (s, 1H_Ar_), 6.81 (d, 2H_Ar_, *J* = 2.0 Hz), 7.00 (d, 2H_Ar_, *J* = 2.0 Hz), 7.70 (d, 1H_Ar_, *J* = 9.2 Hz), 7.97 (dd, 1H_Ar_, *J*_1_ = *J*_2_ = 2.4 Hz), 8.39 (d, 1H_Ar_, *J* = 2.0 Hz), 8.98 (s, 1H_Ar_), 10.15 (s, 1H_N–H_), 11.12 (s, 1H_N–H_); ^13^C NMR (100 MHz, DMSO-d_6_) *δ* (ppm) = 12.6, 55.6, 96.8, 114.8, 118.1, 118.8, 121.4, 124.3, 129.8, 130.0, 132.2, 136.6, 147.3, 156.4, 157.2, 157.7, 159.9, 160.1, 170.9. Anal. Calcd. for C_21_H_17_N_3_O_7_S, %: C, 55.38; H, 3.76; N, 9.23. Found, %: C, 55.52; H, 3.65; N, 9.45.

#### 2-Oxo-6-o-tolylsulfamoyl-2H-chromene-3-carboxylic acid (5-methyl-isoxazol-3-yl)-amide (4e)

Cream Solid; M.p = 224–226 °C; R_f_ = 0.24 (EA: *n*-Hex; 35:65); IR (KBr, cm^−1^) = 3302 (N–H), 1740, 1687 (C=O), 1331, 1158 (SO_2_); ^1^H NMR (400 MHz, DMSO-d_6_) *δ* (ppm) = 2.06 (s, 3H_CH3_), 2.44 (s, 3H_CH3_), 6.79 (s, 1H_Ar_), 6.94 (m, 1H_Ar_), 7.12 (m, 3H_Ar_), 7.73 (d, 1H_Ar_, *J* = 8.8 Hz), 7.97 (dd, 1H_Ar_, *J*_1_ = *J*_2_ = 2.0 Hz), 8.37 (d, 1H_Ar_, *J* = 2.0 Hz), 8.99 (s, 1H_Ar_), 9.84 (s, 1H_N–H_), 11.13 (s, 1H_N–H_); ^13^C NMR (100 MHz, DMSO-d_6_) *δ* (ppm) = 12.6, 18.2, 96.8, 118.2, 118.8, 121.6, 126.9, 127.0, 127.2, 129.5, 131.3, 132.2, 134.8, 134.9, 137.8, 147.3, 156.4, 157.7, 159.9, 160.2, 170.9. Anal. Calcd. for C_21_H_17_N_3_O_6_S, %: C, 57.4; H, 3.90; N, 9.54. Found, %: C, 57.15; H, 3.81; N, 9.63.

#### 2-Oxo-6-p-tolylsulfamoyl-2H-chromene-3-carboxylic acid (5-methyl-isoxazol-3-yl)-amide (4f.)

Light Cream Solid; M.p = 236–238 °C; R_f_ = 0.28 (EA: *n*-Hex; 35:65); IR (KBr, cm^−1^) = 3207 (N–H), 1716, 1673 (C=O), 1338, 1158 (SO_2_); ^1^H NMR (400 MHz, DMSO-d_6_) *δ* (ppm) = 2.19 (s, 3H_CH3_), 2.45 (s, 3H_CH3_), 6.79 (s, 1H_Ar_), 7.00 (d, 2H_Ar_, *J* = 8.4 Hz), 7.05 (d, 2H_Ar_, *J* = 8.4 Hz), 7.70 (d, 1H_Ar_, *J* = 8.8 Hz), 8.01 (dd, 1H_Ar_, *J*_1_ = *J*_2_ = 2.0 Hz), 8.45 (d, 1H_Ar_, *J* = 1.6 Hz), 8.98 (d, 1H_Ar_, *J* = 2.0 Hz), 10.34 (s, 1H_N–H_), 11.11 (s, 1H_N–H_); ^13^C NMR (100 MHz, DMSO-d_6_) *δ* (ppm) = 12.6, 20.7, 96.8, 118.2, 118.8, 121.3, 121.5, 129.9, 130.1, 132.1, 134.2, 134.9, 136.6, 147.2, 156.4, 157.6, 157.7, 159.8, 160.0, 160.1, 170.9. Anal. Calcd. for C_21_H_17_N_3_O_6_S, %: C, 57.4; H, 3.90; N, 9.54. Found, %: C, 57.19; H, 3.77; N, 9.61.

#### 6-(2,4-Dimethyl-phenylsulfamoyl)-2-oxo-2H-chromene-3-carboxylic acid (5-methyl-isoxazol-3-yl)-amide (4g)

Light Cream Solid; M.p = 208–210 °C; R_f_ = 0.32 (EA: *n*-Hex; 35:65); IR (KBr, cm^−1^) = 3281 (N–H), 1722, 1698 (C=O), 1334, 1152 (SO_2_); ^1^H NMR (400 MHz, DMSO-d_6_) *δ* (ppm) = 2.02 (s, 3H_CH3_), 2.21 (s, 3H_CH3_), 2.45 (s, 3H_CH3_), 6.79 (s, 1H_Ar_), 6.81 (s, 1H_Ar_), 6.89 (d, 1H_Ar_, *J* = 8 Hz), 6.98 (s, 1H_Ar_), 7.72 (d, 1H_Ar_, *J* = 8.8 Hz), 7.9 (dd, 1H_Ar_, *J*_1_ = *J*_2_ = 2.0 Hz), 8.35 (d, 1H_Ar_, *J* = 2.0 Hz), 8.99 (s, 1H_Ar_), 9.70 (s, 1H_N–H_), 11.12 (s, 1H_N–H_); ^13^C NMR (100 MHz, DMSO-d_6_) *δ* (ppm) = 12.6, 18.1, 20.9, 96.8, 118.1, 118.8, 121.5, 127.4, 127.4, 129.5, 131.9, 132.1, 132.3, 135.0, 136.6, 137.9, 147.3, 156.4, 157.7, 159.9, 160.2, 170.9. Anal. Calcd. for C_22_H_19_N_3_O_6_S, %: C, 58.27; H, 4.22; N, 9.27. Found, %: C, 58.39; H, 4.30; N, 9.15.

#### 6-(2-Chloro-phenylsulfamoyl)-2-oxo-2H-chromene-3-carboxylic acid (5-methyl-isoxazol-3-yl)-amide (4h)

Dark Cream Solid; M.p = 230–234 °C; R_f_ = 0.32 (EA: *n*-Hex; 35:65); IR (KBr, cm^−1^) = 3275 (N–H), 1740, 1691 (C=O), 1334, 1158 (SO_2_); ^1^H NMR (400 MHz, DMSO-d_6_) *δ* (pm) = 2.44 (s, 3H_CH3_), 6.78 (s, 1H_Ar_), 7.25 (m, 1H_Ar_), 7.31 (d, 1H_Ar_, *J* = 1.6 Hz), 7.32 (dd, 1H_Ar_, *J*_1_ = *J*_2_ = 1.2 Hz), 7.42 (t, 2H_Ar_), 7.73 (d, 1H_Ar_, *J* = 8.8 Hz), 8.03 (dd, 1H_Ar_, *J*_1_ = *J*_2_ = 2.4 Hz), 8.42 (d, 1H_Ar_, *J* = 2.0 Hz), 8.99 (s, 1H_Ar_), 10.29 (s, 1H_N–H_), 11.11 (s, 1H_N–H_); ^13^C NMR (100 MHz, DMSO-d_6_) *δ* (ppm) = 12.6, 96.8, 118.2, 118.8, 121.5, 128.4, 128.6, 129.7, 129.9, 130.4, 132.4, 133.5, 137.6, 147.3, 156.5, 157.7, 159.8, 160.2, 170.9. Anal. Calcd. for C_20_H_14_ClN_3_O_6_S, %: C, 52.24; H, 3.07; N, 9.14. Found, %: C, 52.38; H, 3.14; N, 9.20.

#### 6-(3-Chloro-phenylsulfamoyl)-2-oxo-2H-chromene-3-carboxylic acid (5-methyl-isoxazol-3-yl)-amide (4i)

Cream Solid; M.p = 245–250 °C; R_f_ = 0.2 (EA: *n*-Hex; 35:65); IR (KBr, cm^−1^) = 3206 (N–H), 1716, 1680 (C=O), 1345, 1154 (SO_2_); ^1^H NMR (400 MHz, DMSO-d_6_) *δ* (ppm) = 2.44 (s, 3H_CH3_), 6.78 (s, 1H_Ar_), 7.11 (m, 2H_Ar_), 7.17 (s, 1H_Ar_), 7.27 (t, 1H_Ar_), 7.73 (d, 1H_Ar_, *J* = 8.8 Hz), 8.0 (dd, 1H_Ar_, *J*_1_ = *J*_2_ = 2.0 Hz), 8.55 (s, 1H_Ar_), 9.0 (d, 1H_Ar_, *J* = 2.8 Hz), 10.82 (s, 1H_N–H_), 11.10 (s, 1H_N–H_); ^13^C NMR (100 MHz, DMSO-d_6_) *δ* (ppm) = 12.6, 96.8, 118.5, 118.6, 119.0, 119.8, 121.4, 124.5, 130.0, 131.5, 132.0, 134.0, 136.2, 139.3, 147.3, 156.6, 157.7, 159.9, 160.0, 170.9. Anal. Calcd. for C_20_H_14_ClN_3_O_6_S, %: C, 52.24; H, 3.07; N, 9.14. Found, %: C, 52.43; H, 3.18; N, 9.28.

##### 6-(4-Chloro-phenylsulfamoyl)-2-oxo-2H-chromene-3-carboxylic acid (5-methyl-isoxazol-3-yl)-amide (4j)

Light Yellow Solid; M.p = 230–233 °C, R_f_ = 0.16 (EA: *n*-Hex; 35:65); IR (KBr, cm^−1^) = 3275 (N–H), 1730, 1679 (C=O), 1339, 1155 (SO_2_); ^1^H NMR (400 MHz, DMSO-d_6_) *δ* (ppm) = 2.44 (s, 3H_CH3_), 6.77 (s, 1H_Ar_), 7.16 (d, 2H_Ar_, *J* = 2.0 Hz), 7.31 (d, 2H_Ar_, *J* = 2.0 Hz), 7.71 (d, 1H_Ar_, *J* = 8.8 Hz), 8.04 (dd, 1H_Ar_, *J*_1_ = *J*_2_ = 2.4 Hz), 8.49 (d, 1H_Ar_, *J* = 2.0 Hz), 9.00 (s, 1H_Ar_), 10.67 (s, 1H_N–H_), 11.10 (s, 1H_N–H_); ^13^C NMR (100 MHz, DMSO-d_6_) *δ* (ppm) = 12.6, 96.8, 118.4, 118.9, 121.5, 122.3, 129.0, 130.0, 132.0, 136.2, 136.7, 147.3, 156.6, 157.7, 159.8, 160.1, 170.9. Anal. Calcd. for C_20_H_14_ClN_3_O_6_S, %: C, 52.24; H, 3.07; N, 9.14. Found, %: C, 52.49.02; H, 3.11; N, 9.27.

##### 6-(Ethyl-phenyl-sulfamoyl)-2-oxo-2H-chromene-3-carboxylic acid (5-methyl-isoxazol-3-yl)-amide (4k)

Dark Cream Solid; M.p = 220–222 °C; R_f_ = 0.44 (EA: *n*-Hex; 35:65); IR (KBr, cm^−1^) = 3253 (N–H), 1730, 1684 (C=O), 1350, 1146 (SO_2_); ^1^H NMR (400 MHz, DMSO-d_6_) *δ* (ppm) = 1.0 (t, 3H_CH3_), 2.45 (s, 3H_CH3_), 3.6 (dd, 2H_CH2_, *J* = 7.2 Hz), 6.80 (s, 1H_Ar_), 7.09 (m, 2H_Ar_), 7.35 (m, 3H_Ar_), 7.70 (d, 1H_Ar_, *J* = 8.8 Hz), 7.82 (dd, 1H_Ar_, *J*_1_ = *J*_2_ = 2.4 Hz), 8.44 (d, 1H_Ar_, *J* = 1.6 Hz), 9.03 (s, 1H_Ar_), 11.14 (s, 1H_N–H_); ^13^C NMR (100 MHz, DMSO-d_6_) *δ* (ppm) = 12.6, 14.3, 45.9, 96.8, 118.0, 119.1, 121.4, 128.6, 129.19, 129.7, 130.3, 132.8, 135.1, 138.5, 147.5, 156.6, 157.7, 159.9, 160.2, 170.9. Anal. Calcd. for C_22_H_19_N_3_O_6_S, %: C, 58.27; H, 4.22; N, 9.27. Found, %: C, 58.89; H, 4.30; N, 8.95.

##### 6-Dipropylsulfamoyl-2-oxo-2H-chromene-3-carboxylic acid (5-methyl-isoxazol-3-yl)-amide (4l)

White Solid; M.p = 200–204 °C; R_f_ = 0.48 (EA: *n*-Hex; 35:65); IR (KBr, cm^−1^) = 3241 (N–H), 2971, 1732 (C=O), 1682 (C=O), 1339, 1145 (SO_2_); ^1^H NMR (400 MHz, DMSO-d_6_) *δ* (ppm) = 0.82 (t, 6H_CH3_), 1.48 (m, 4H_CH2_), 2.45 (s, 3H_CH3_), 3.06 (t, 4H_CH2_), 6.80 (s, 1H_Ar_), 7.73 (d, 1H_Ar_, *J* = 8.8 Hz), 8.13 (dd, 1H_Ar_, *J*_1_ = *J*_2_ = 2.4 Hz), 8.58 (d, 1H_Ar_, *J* = 2.4 Hz), 9.05 (s, 1H_N–H_), 11.14 (s, 1H_N–H_); ^13^C NMR (100 MHz, DMSO-d_6_) *δ* (ppm) = 11.4, 12.6, 22.2, 50.3, 96.8, 118.1, 119.1, 121.25, 129.9, 132.4, 136.7, 147.6, 156.3, 157.7, 160.0, 160.1, 170.9. Anal. Calcd. for C_20_H_23_N_3_O_6_S, %: C, 55.42; H, 5.35; N, 9.69. Found, %: C, 54.96; H, 5.42; N, 9.10.

### Spectral data of the synthesized products

All of the spectral data were placed in the supplementary file.

### Antibacterial activity

#### Disk diffusion method

All of the synthesized products were evaluated for their antibacterial property against *Escherichia coli* (*E. coli* ATCC 25922) as Gram-negative and *Staphylococcus aureus* (*S*. *aureus* ATCC 25923) as Gram-positive bacteria using disk diffusion method. The basic solutions of synthesized sulfonamide hybrids (**9a–l**) were prepared with the concentration of 5 mg/mL in pure DMSO. Then these solutions were used to provide serial dilution of compounds with reduced concentrations of 2.5, 1.25, 0.62 and 0.31 mg/mL. Then 50 µl of each concentration was added to sterile paper blank disk and were dried under laminar air flow BL1 at room temperature for 15 min. After that, the tested bacteria were transferred to the sterile tubes containing 4 to 5 mL Nutrient Broth and were incubated at 37 ºC until the turbidity was seen. Their density adjustment has been performed to 0.5 McFarland (equal to 1.5 × 10^8^/ml of bacteria) with sterile saline. After performing autoclave sterilization, nutrient was cultured to Petri dishes for giving a depth of 6 mm. Then, the bacteria expanded on the agar surface and incubated for further 24 h at 37 ºC. The zone of inhibition for each disk was measured in millimeters that indicate how much the tested compounds inhibit the microorganism’s growth.

### Minimum inhibitory concentration (MIC)

The antibacterial activity of the synthesized compounds were applied in vitro against Gram-negative bacteria *E. coli* (ATCC 25922) and Gram-positive bacteria *S. aureus* (ATCC 25923) by broth microdilution method. Muller-Hinton agar was used to make double dilutions of the research compounds and reference drugs. To prepare the samples, 10 mg of each test compound was dissolved in 1 ml of dimethyl sulfoxide (DMSO) as a stock solution and the tested compounds and reference drugs were prepared in Mueller Hinton broth. A second 1% of the DMSO and 1024 µg/ml of all compounds stock were created based on the CLSI standard. Further progressive serial dilution of the compounds were applied to gain the require concentration of 512, 256, 128, 64, 32, 16, 8, 4, 2, 1 µg/ml. A concentration of 1 × 10^5^ CFU was obtained by diluting the bacterial suspension with sterile saline. Each test was performed with triplicate. The MIC was the lowest concentration of the synthesized compounds that produce no growth on the plate. A control solvent of the DMSO with cultured bacteria in the microplate was used to ensure that 1% of the DMSO.

## Supplementary Information

Supplementary Tables.

Supplementary Figures.
