# Supplementary material for: Synthesis of some novel coumarin isoxazol sulfonamide hybrid compounds, 3D-QSAR studies, and antibacterial evaluation
Source: Sci Rep. 2021 Oct 11;11:20088. doi: 10.1038/s41598-021-99618-w (PMC8505453; doi:10.1038/s41598-021-99618-w)
Supplement: Supplementary file 2 — Supplementary Figures. [file 41598_2021_99618_MOESM2_ESM.docx]

SUPPLEMENTARY INFORMATION

**Synthesis of Some Novel Coumarin Isoxazol Sulfonamide Hybrid Compounds, 3D-QSAR Studies, and Antibacterial Evaluation**

**Spectral data of the synthesized products:**

**
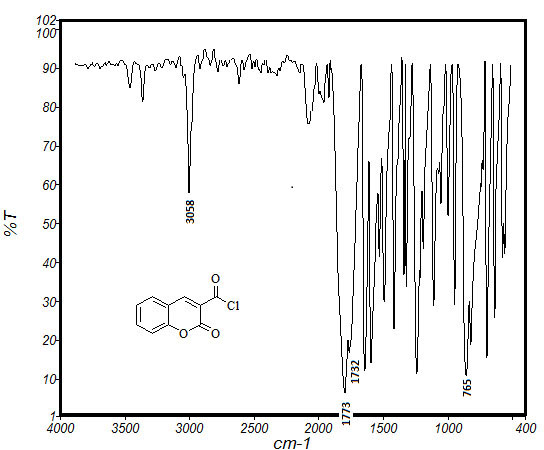
**

**Figure S1. IR spectra of 2-oxo-2H-chromene-3-carbonyl chloride (6)**

**
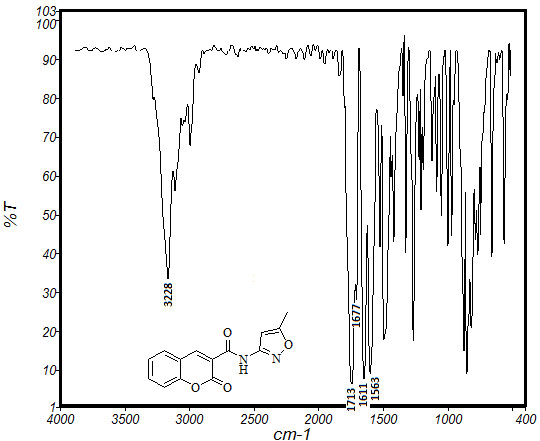
**

**Figure S2. IR spectra of N-(5-methylisoxazol-3-yl)-2-oxo-2H-chromene-3-carboxamide (7)**

**
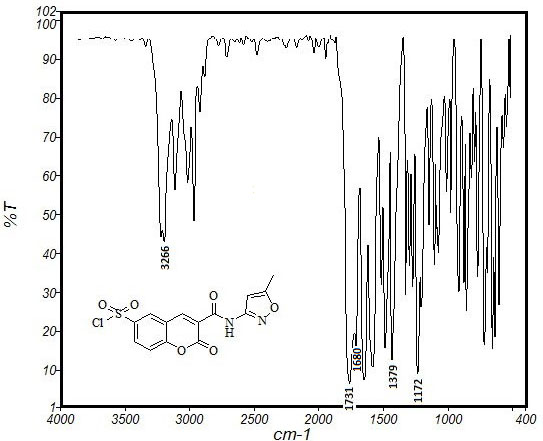
**

**Figure S3. IR spectra of 3-((5-methylisoxazol-3-yl) carbamoyl)-2-oxo-2H-chromene-6-sulfonyl chloride (8)**

| **Figure S4^. 13^C NMR spectra of 3-((5-methylisoxazol-3-yl)carbamoyl)-2-oxo-2H-chromene-6-sulfonyl chloride (8)** | 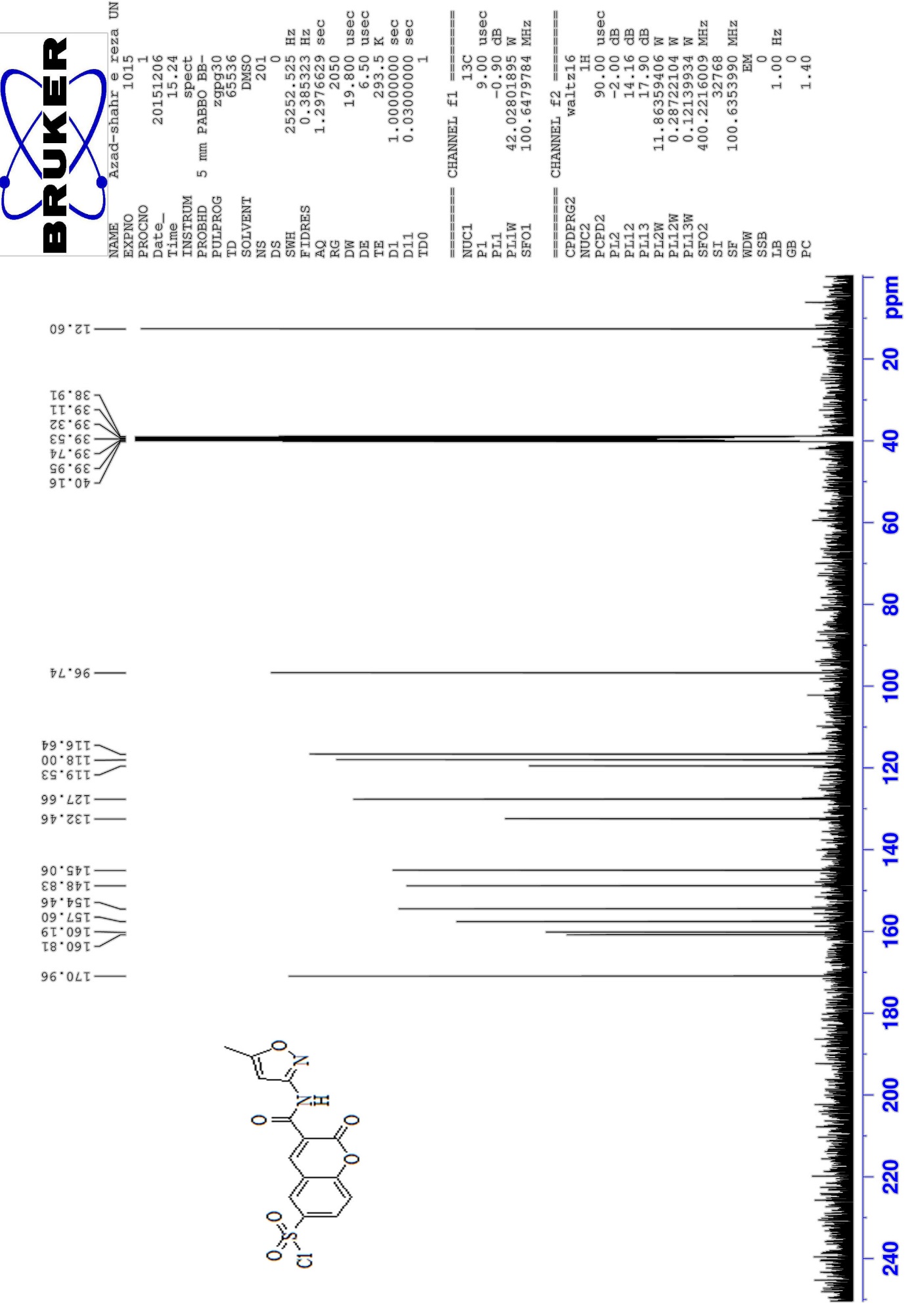 |
| --- | --- |
| **Figure S5. ^1^H NMR spectra of 3-((5-methylisoxazol-3-yl)carbamoyl)-2-oxo-2H-chromene-6-sulfonyl chloride (8)** | 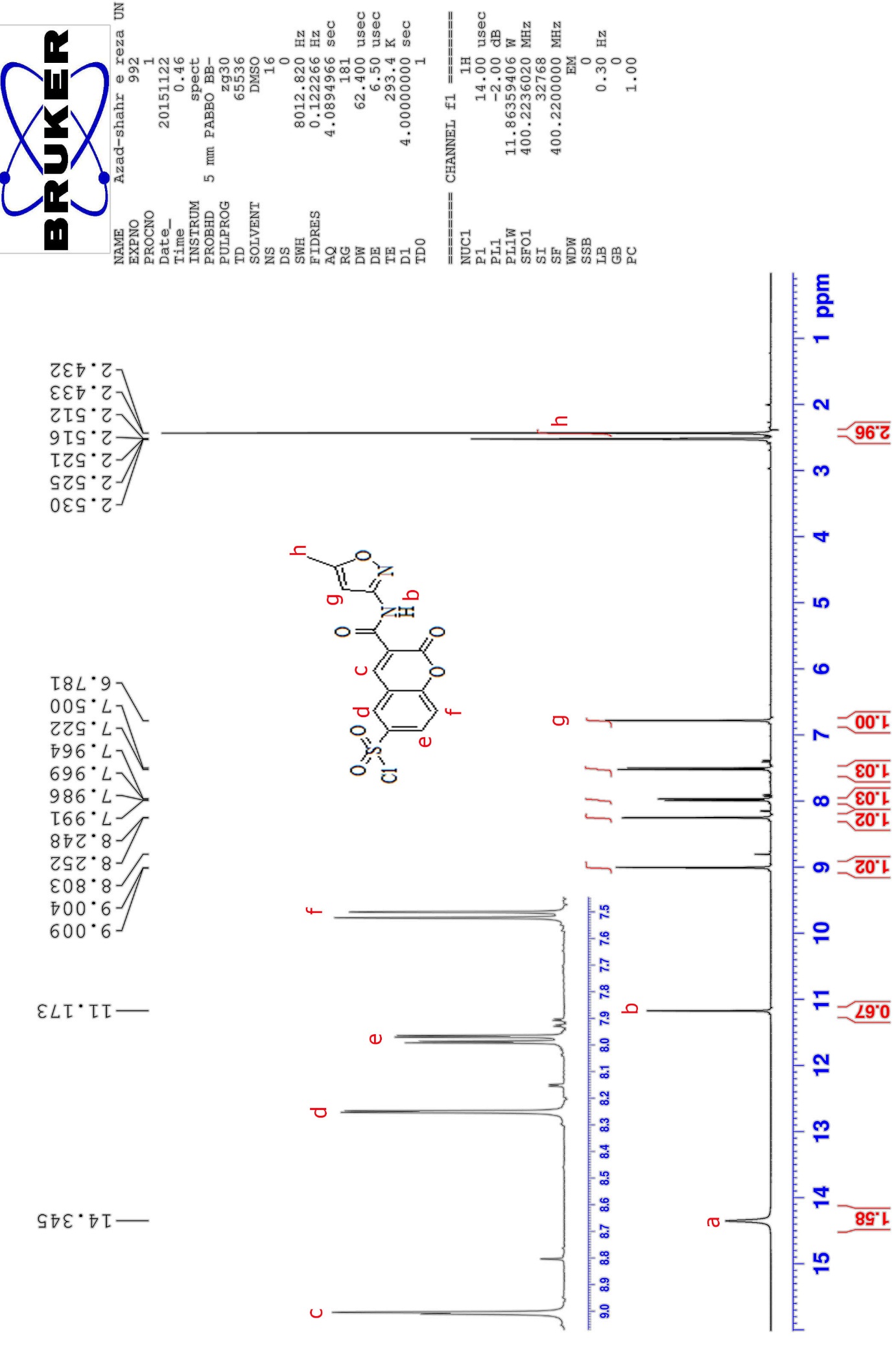 |

**
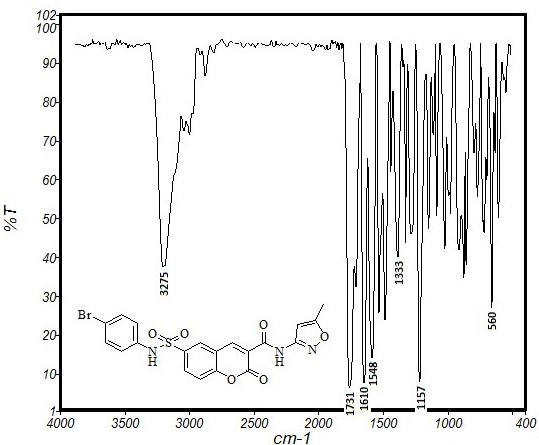
**

**Figure S6. IR spectra of 6-(N-(4-bromophenyl)sulfamoyl)-N-(5-methylisoxazol-3-yl)-2-oxo-2H-chromene-3-carboxamide (*9a*)**

| **Figure S7. ^13^C NMR spectra of 6-(N-(4-bromophenyl)sulfamoyl)-N-(5-methylisoxazol-3-yl)-2-oxo-2H-chromene-3-carboxamide (*9a*)** | 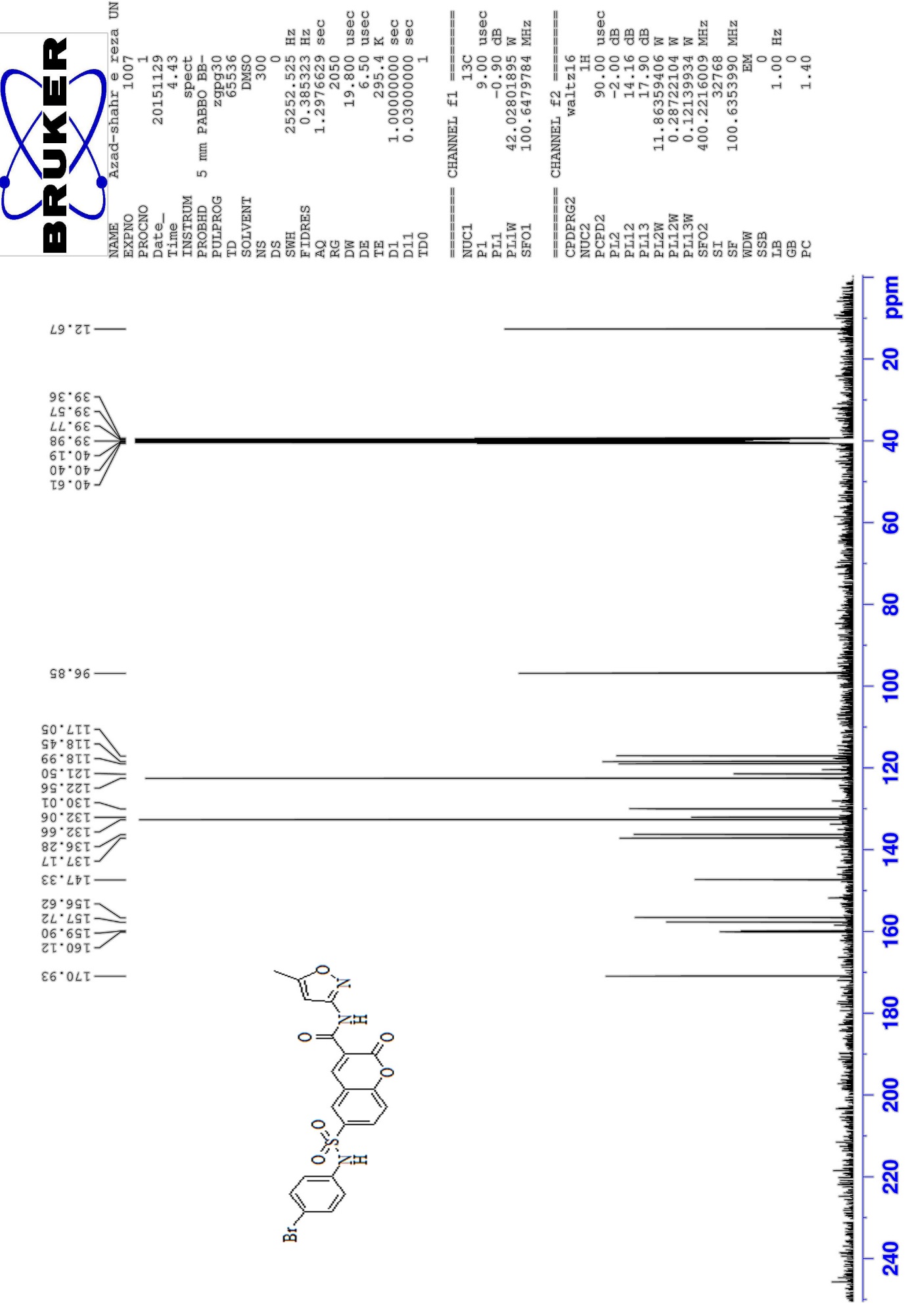 |
| --- | --- |
| **Figure S8. ^1^H NMR spectra of 6-(N-(4-bromophenyl)sulfamoyl)-N-(5-methylisoxazol-3-yl)-2-oxo-2H-chromene-3-carboxamide (*9a*)** | 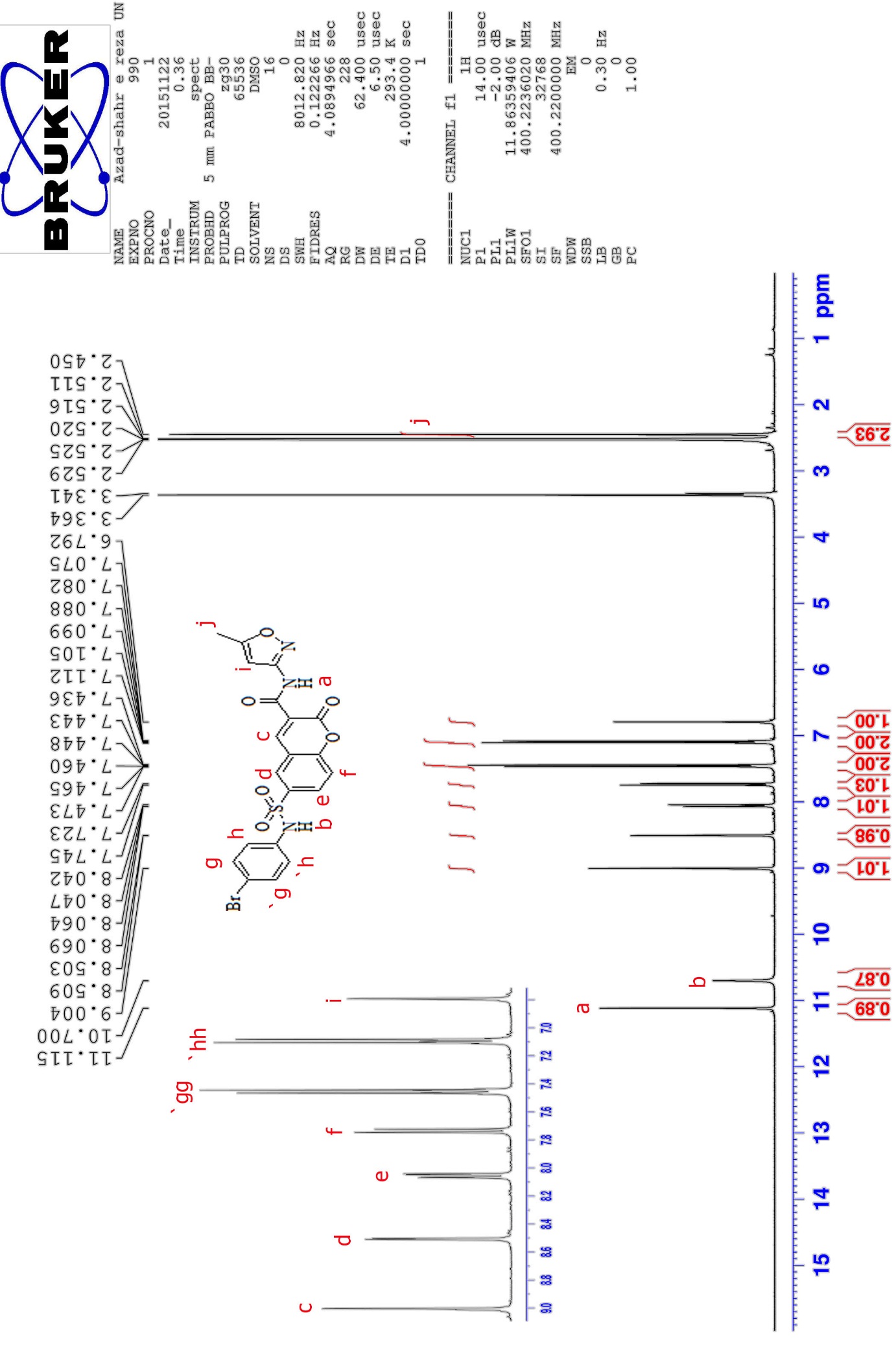 |


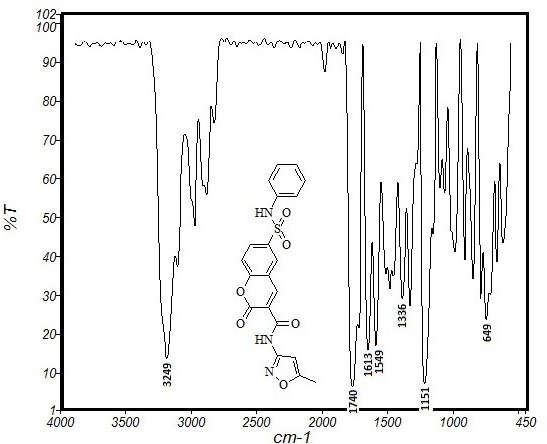


**Figure S9. IR spectra of 6-(N-(phenyl) sulfamoyl)-N-(5-methylisoxazol-3-yl)-2-oxo-2H-chromene-3 carboxamide (*9b*)**

| **Figure 10. ^13^C NMR spectra of 6-(N-(phenyl)sulfamoyl)-N-(5-methylisoxazol-3-yl)-2-oxo-2H-chromene-3 carboxamide (*9b*)** | 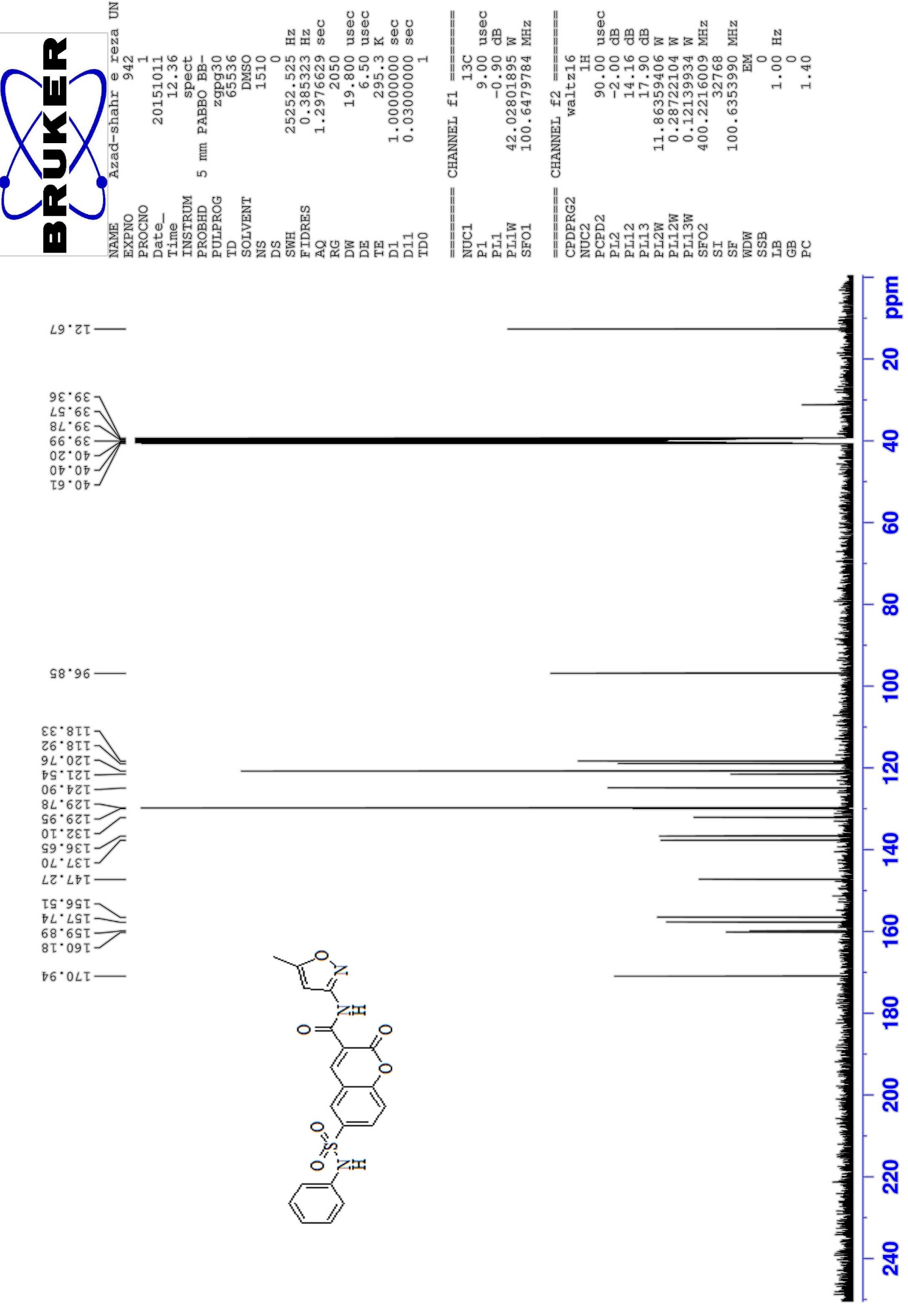 |
| --- | --- |
| **Figure S11. ^1^H NMR spectra of 6-(N-(phenyl)sulfamoyl)-N-(5-methylisoxazol-3-yl)-2-oxo-2H-chromene-3 carboxamide (*9b*)** | 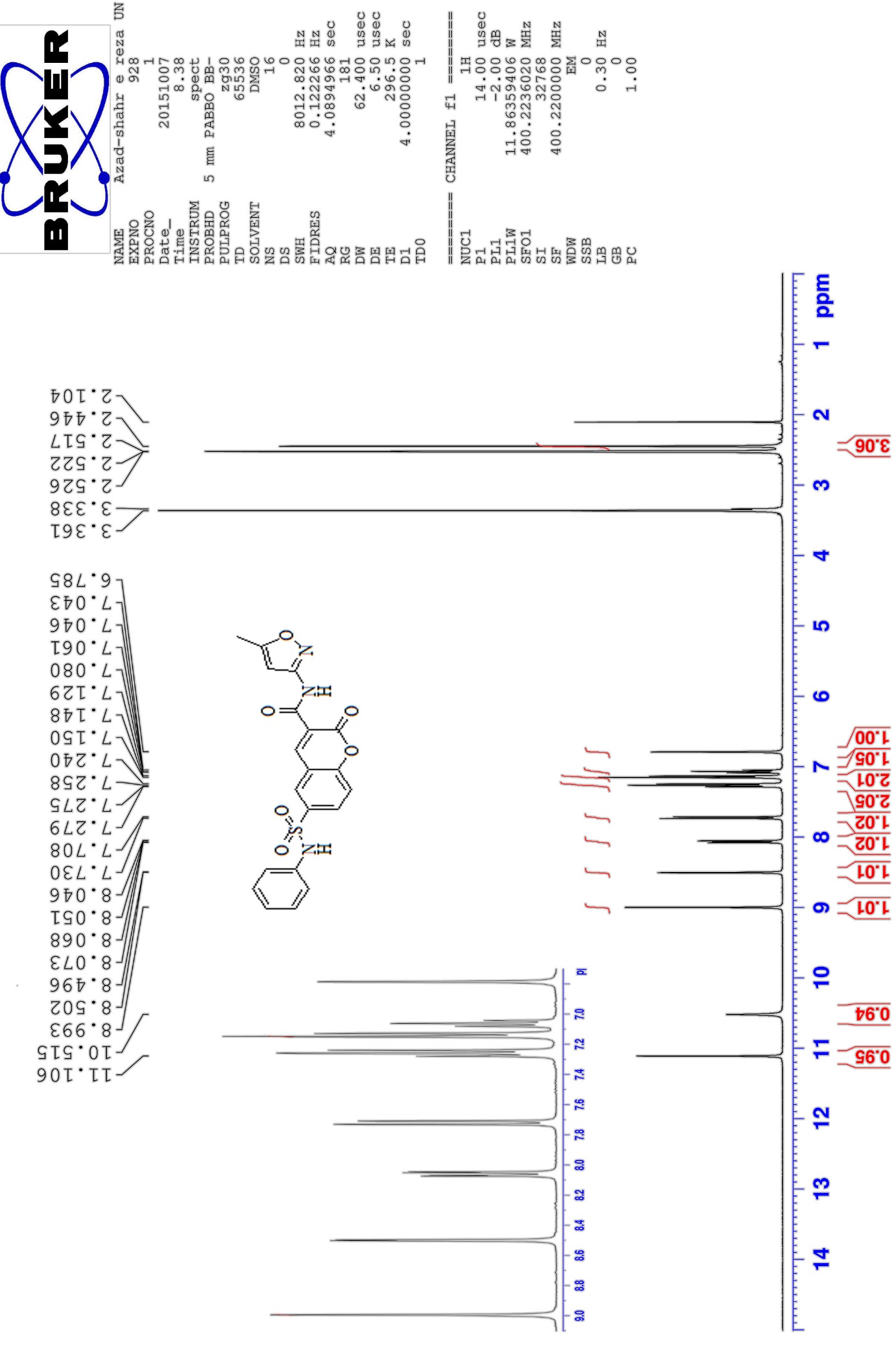 |


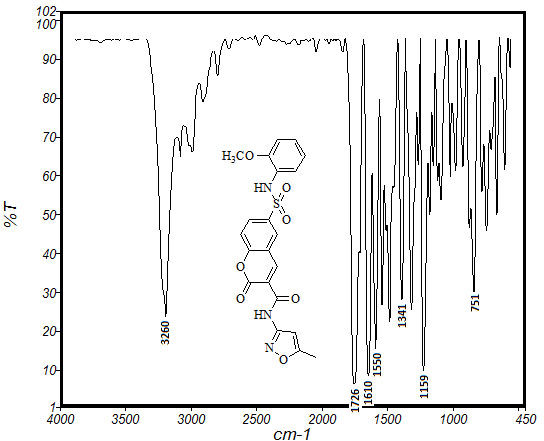


**Figure S12. IR spectra of 6-(2-Methoxy-phenylsulfamoyl)-2-oxo-2H-chromene-3-carboxylic acid (5-methyl-isoxazol-3-yl)-amide (*9c*)**

| **Figure S13. ^13^C NMR spectra of 6-(2-Methoxy-phenylsulfamoyl)-2-oxo-2H-chromene-3-carboxylic acid (5-methyl-isoxazol-3-yl)-amide (*9c*)** | 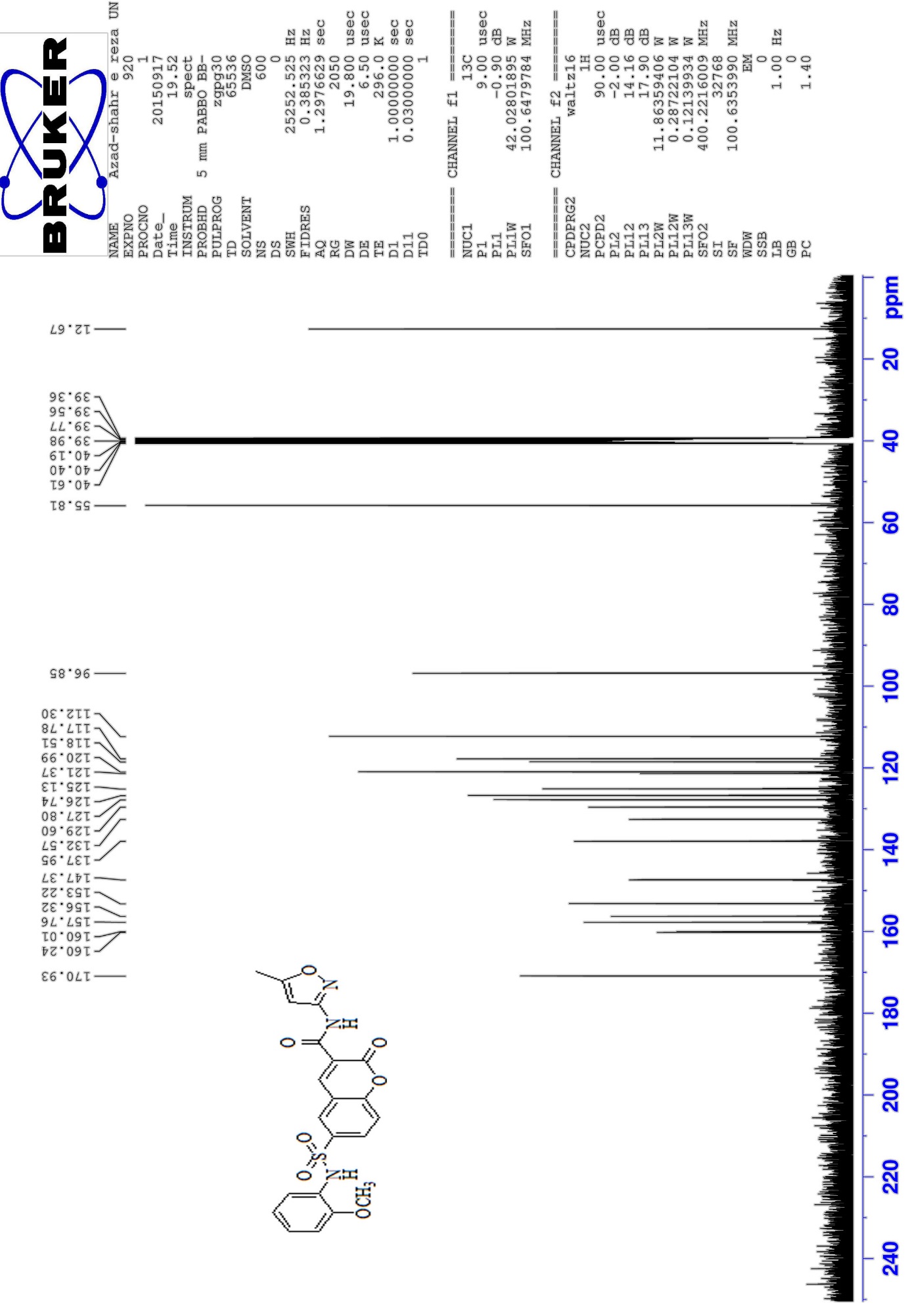 |
| --- | --- |
| **Figure S14. ^1^H NMR spectra of 6-(2-Methoxy-phenylsulfamoyl)-2-oxo-2H-chromene-3-carboxylic acid (5-methyl-isoxazol-3-yl)-amide (*9c*)** | 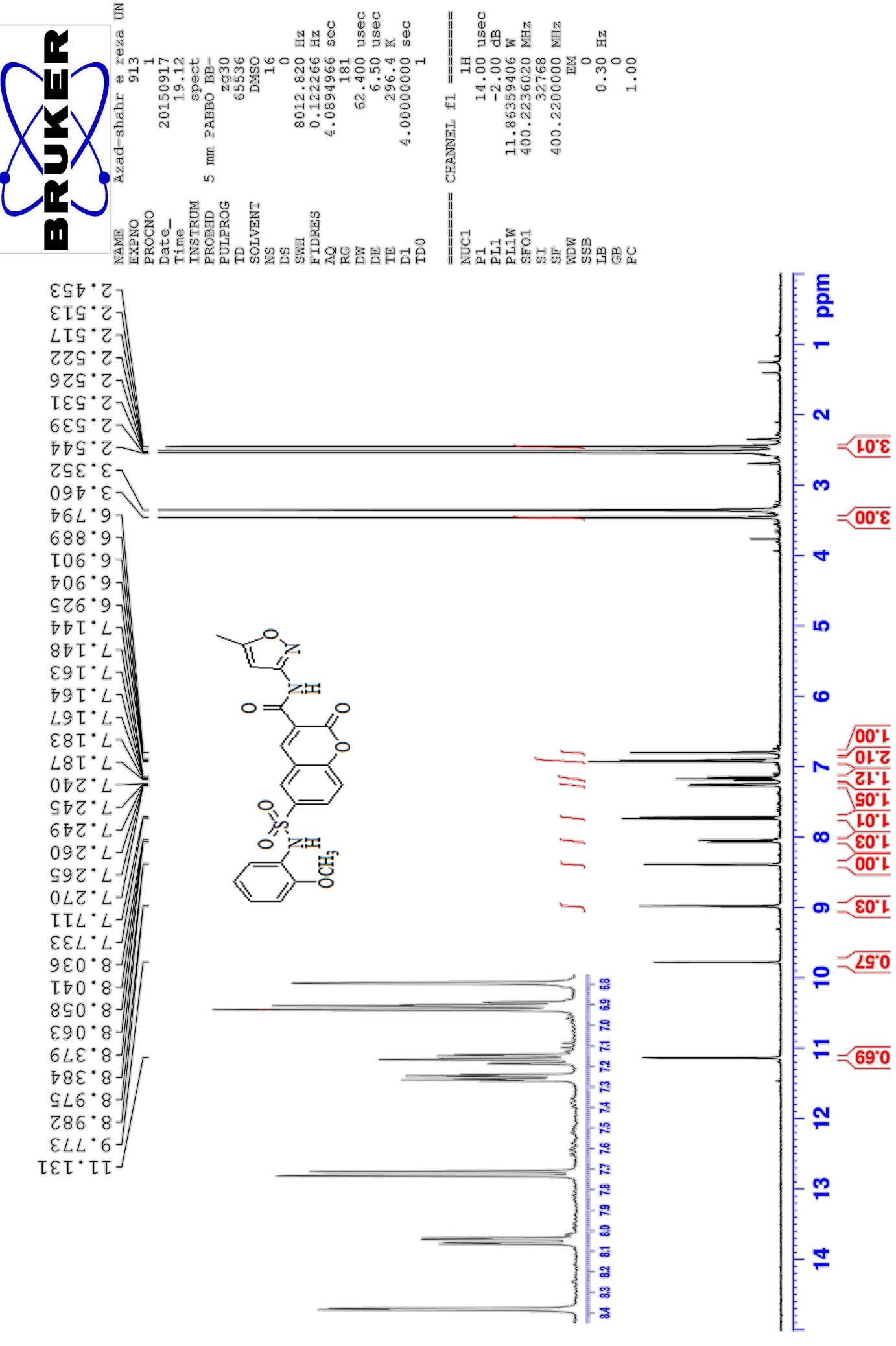 |


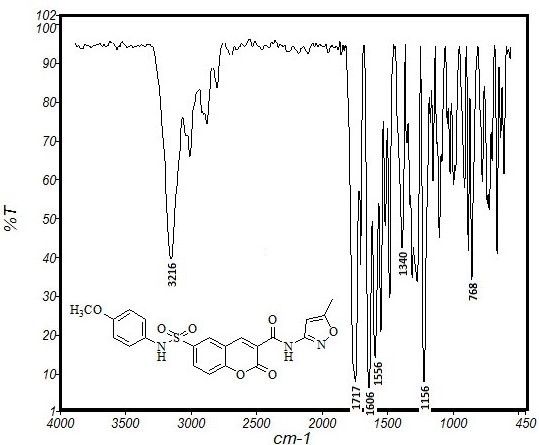


**Figure S15. IR spectra of 6-(4-Methoxy-phenylsulfamoyl)-2-oxo-2H-chromene-3-carboxylic acid (5-methyl-isoxazol-3-yl)-amide (*9d*)**

| **Figure S16. ^13^C NMR spectra of 6-(4-Methoxy-phenylsulfamoyl)-2-oxo-2H-chromene-3-carboxylic acid (5-methyl-isoxazol-3-yl)-amide (*9d*)** | 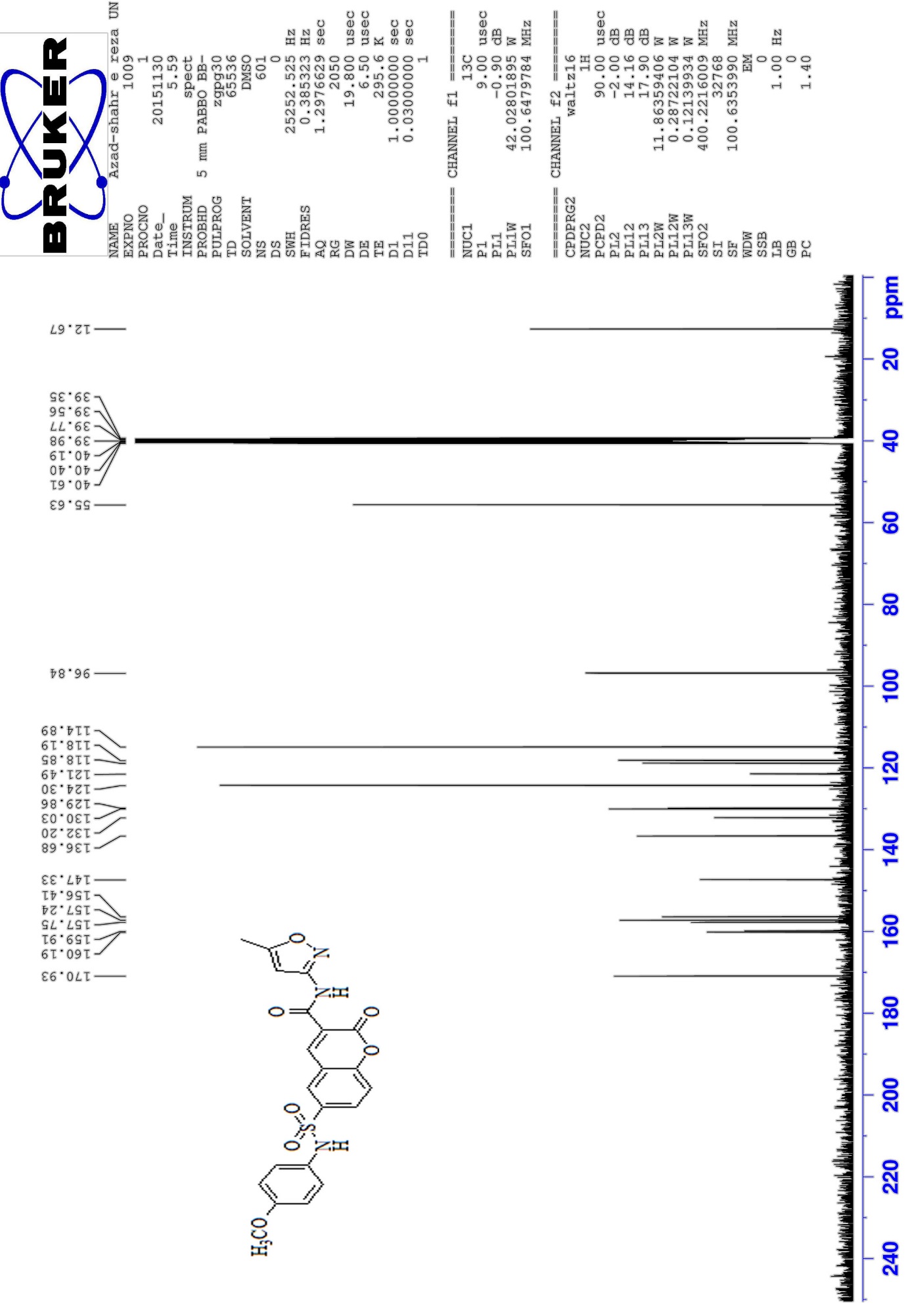 |
| --- | --- |
| **Figure S17. ^1^H NMR spectra of 6-(4-Methoxy-phenylsulfamoyl)-2-oxo-2H-chromene-3-carboxylic acid (5-methyl-isoxazol-3-yl)-amide (*9d*)** | 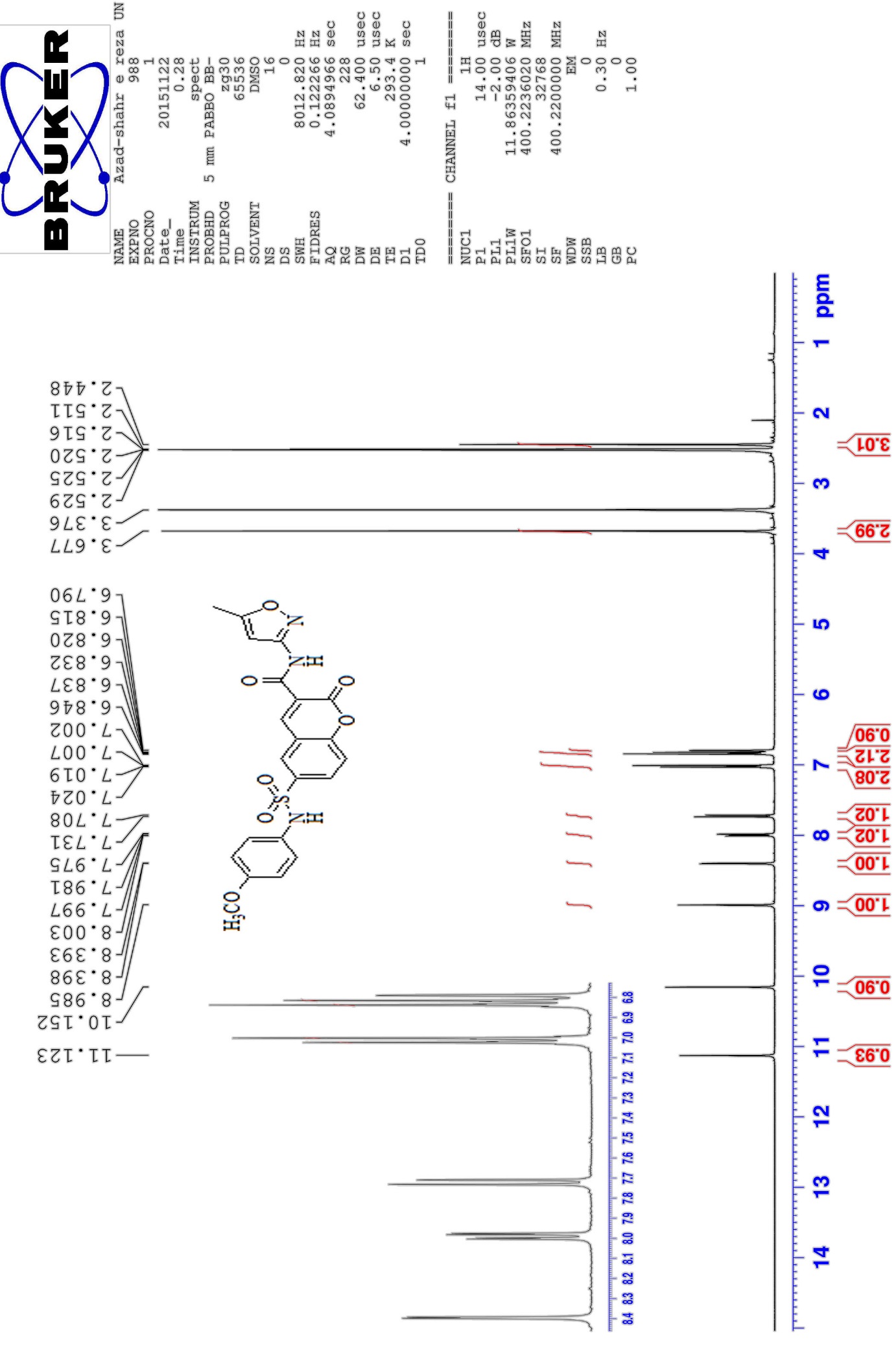 |


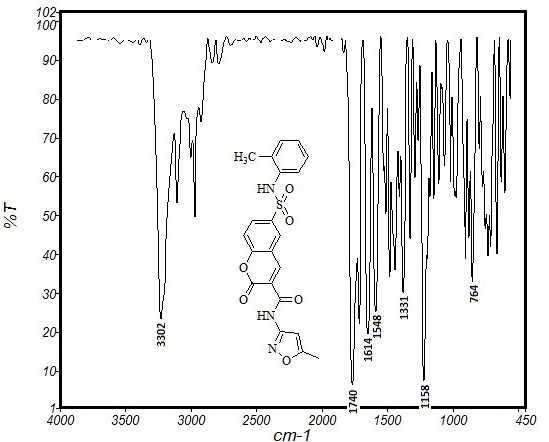


**Figure S18. IR spectra of 2-Oxo-6-o-tolylsulfamoyl-2H-chromene-3-carboxylic acid (5-methyl-isoxazol-3-yl)-amide (*9e*)**

| **Figure S19. ^13^C NMR spectra of 2-Oxo-6-o-tolylsulfamoyl-2H-chromene-3-carboxylic acid (5-methyl-isoxazol-3-yl)-amide (*9e*)** | 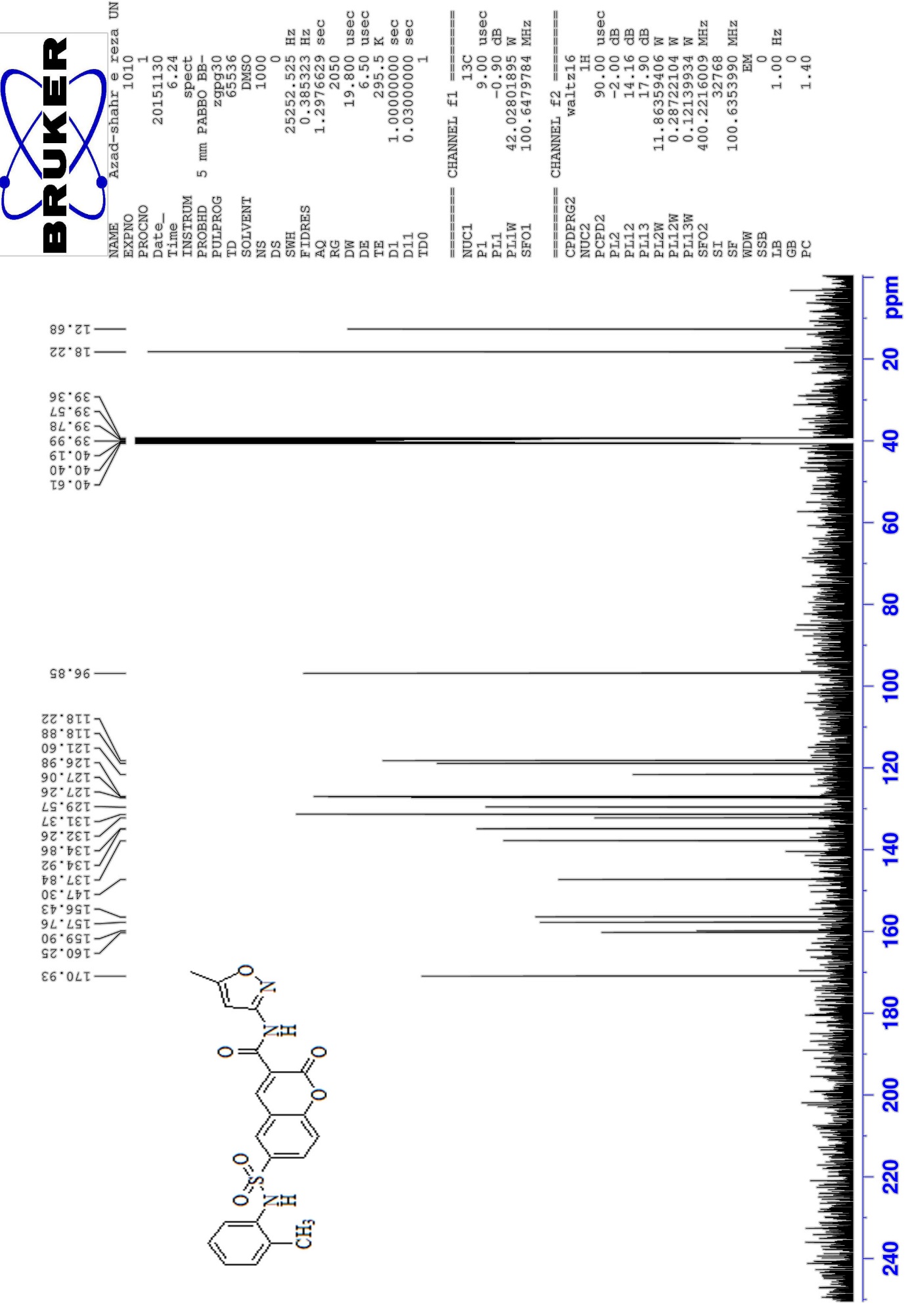 |
| --- | --- |
| **Figure S20. ^1^H NMR spectra of 2-Oxo-6-o-tolylsulfamoyl-2H-chromene-3-carboxylic acid (5-methyl-isoxazol-3-yl)-amide (*9e*)** | 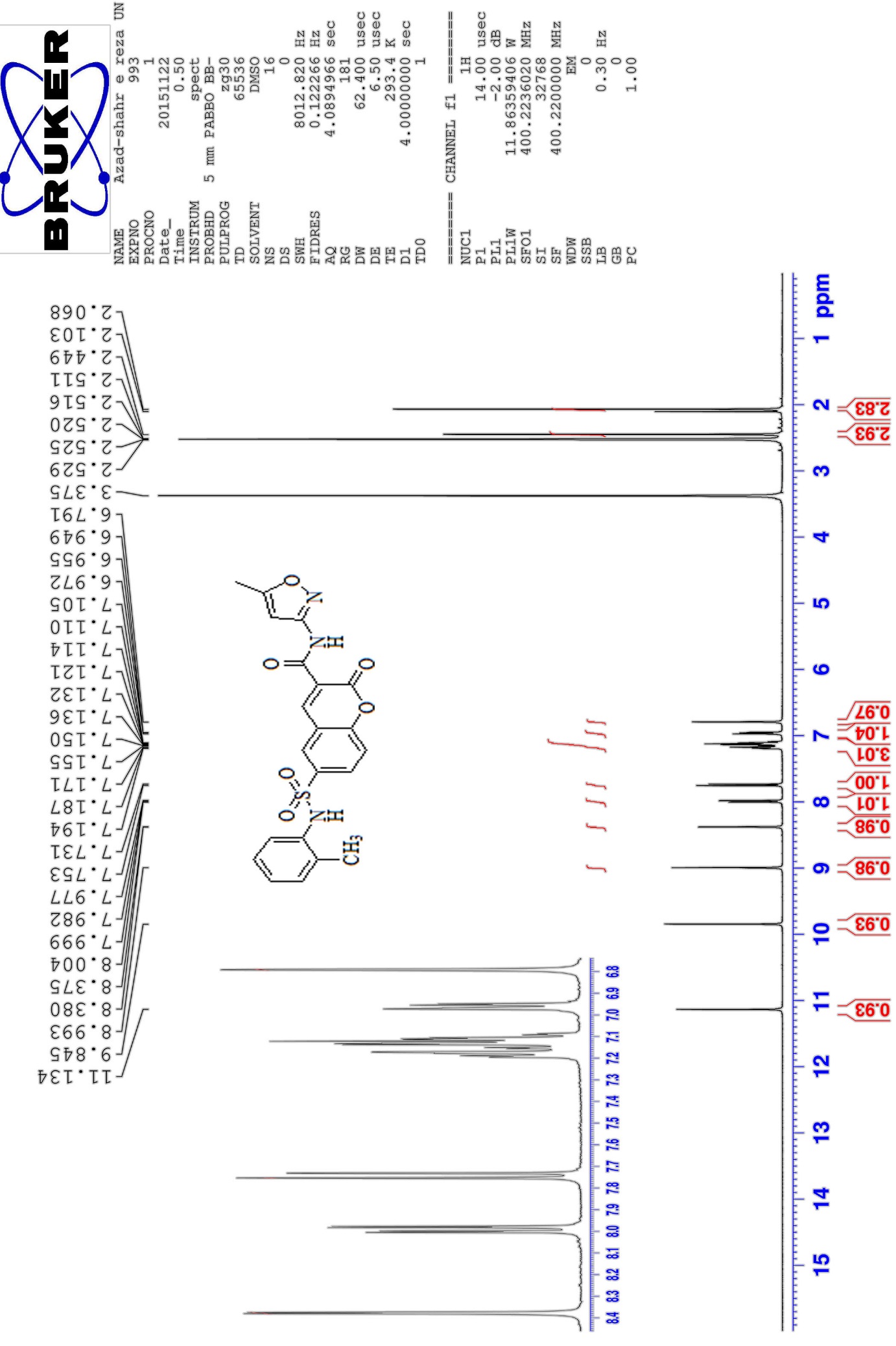 |


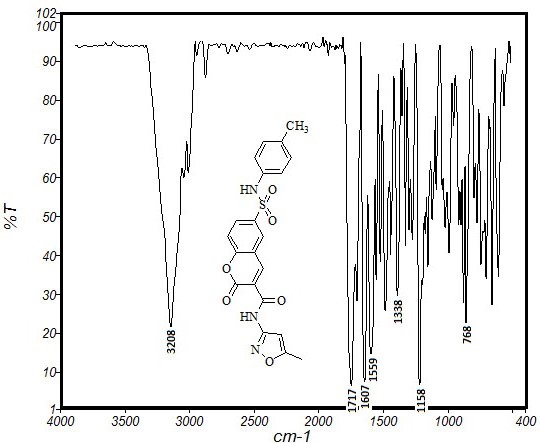


**Figure S21. IR spectra of 2-Oxo-6-p-tolylsulfamoyl-2H-chromene-3-carboxylic acid (5-methyl-isoxazol-3-yl)-amide (*9f*)**

| **Figure S22. ^13^C NMR spectra of 2-Oxo-6-p-tolylsulfamoyl-2H-chromene-3-carboxylic acid (5-methyl-isoxazol-3-yl)-amide (*9f*)** | 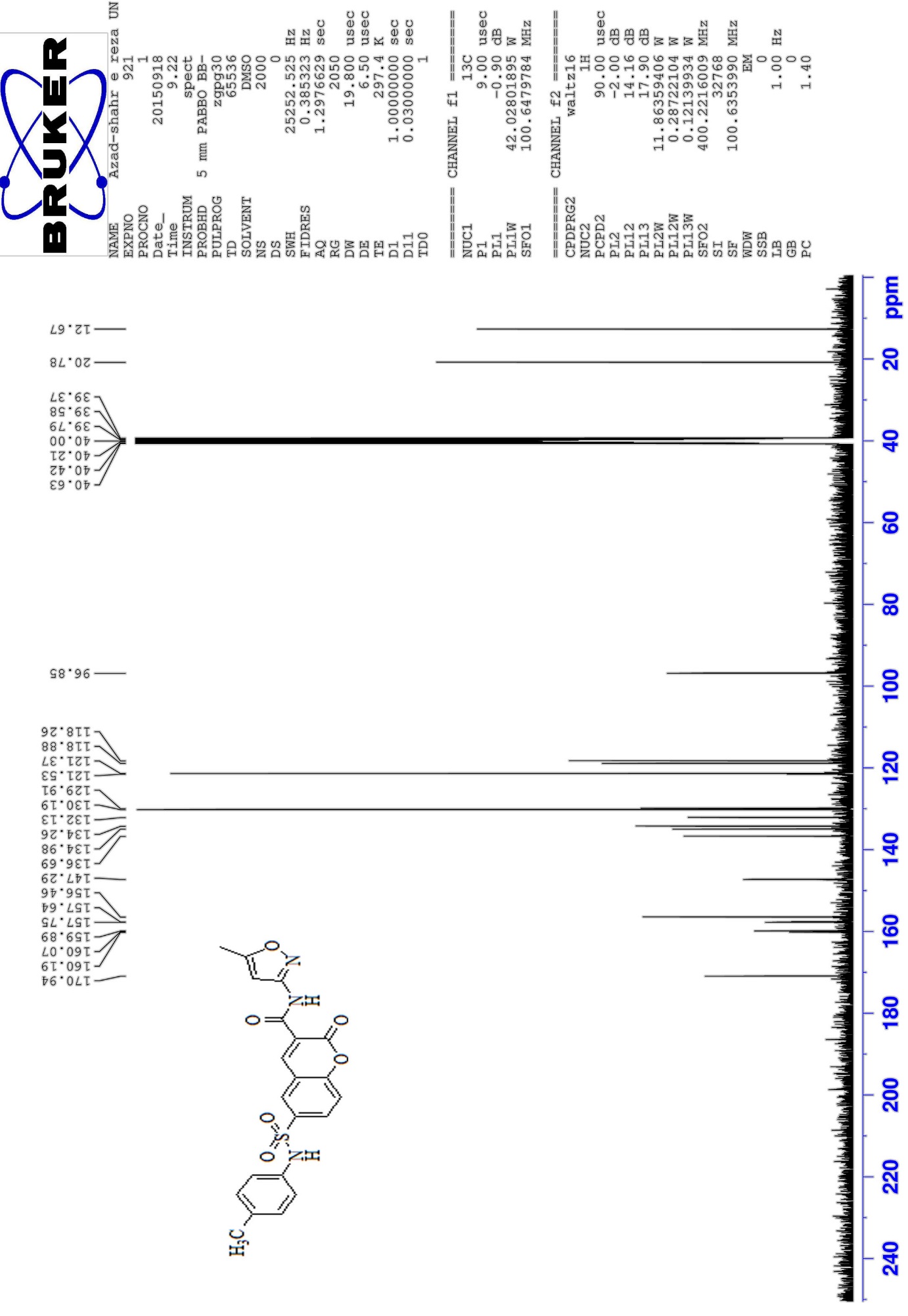 |
| --- | --- |
| **Figure S23. ^1^H NMR spectra of 2-Oxo-6-p-tolylsulfamoyl-2H-chromene-3-carboxylic acid (5-methyl-isoxazol-3-yl)-amide (*9f*)** | 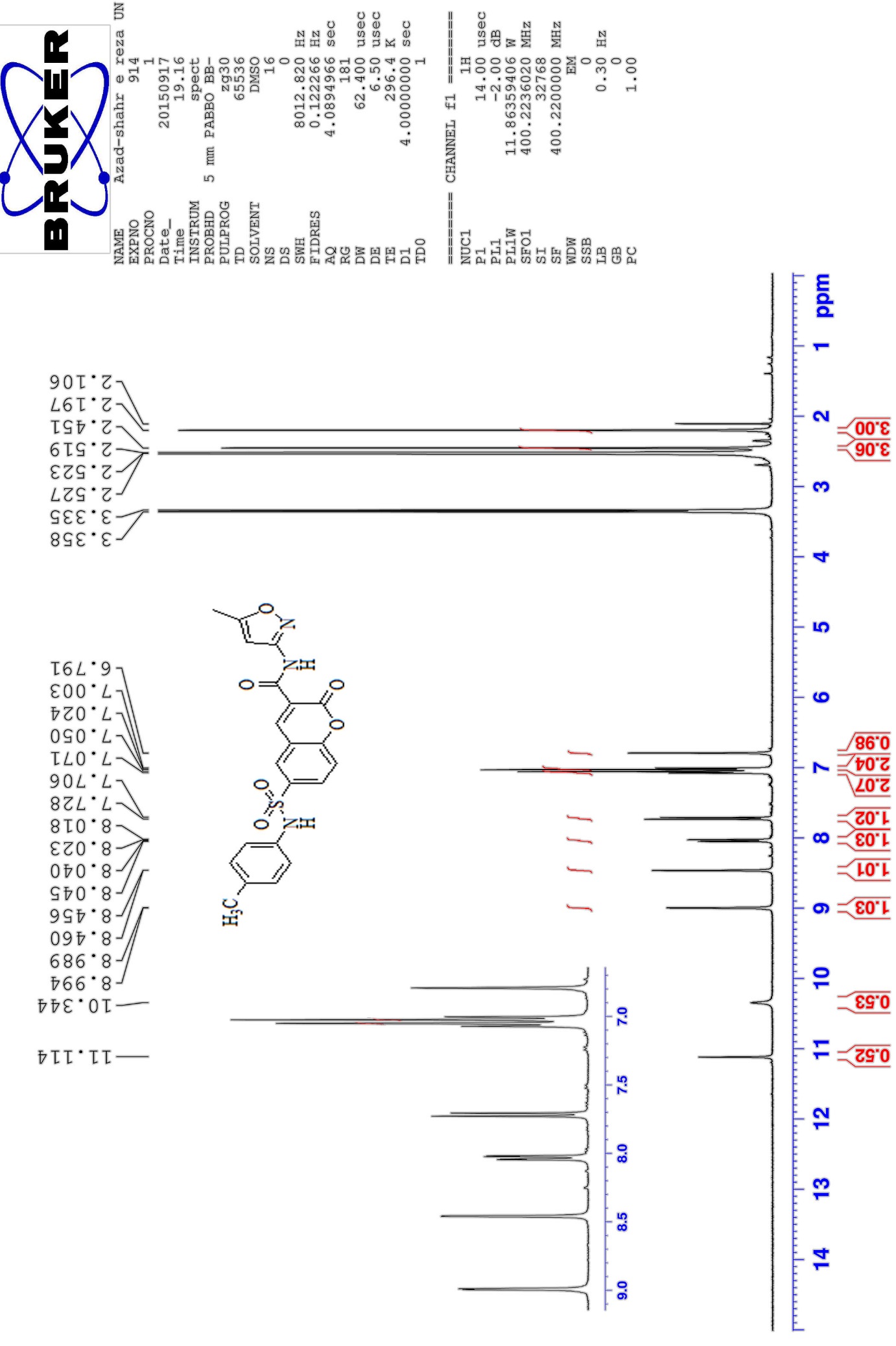 |


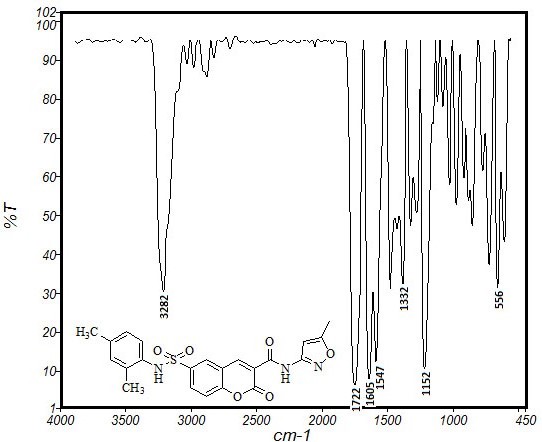


**Figure S24. IR spectra of 6-(2,4-Dimethyl-phenylsulfamoyl)-2-oxo-2H-chromene-3-carboxylic acid (5-methyl-isoxazol-3-yl)-amide (*9g*)**

| **Figure S25. ^13^C NMR spectra of 6-(2,4-Dimethyl-phenylsulfamoyl)-2-oxo-2H-chromene-3-carboxylic acid (5-methyl-isoxazol-3-yl)-amide (*9g*)** | 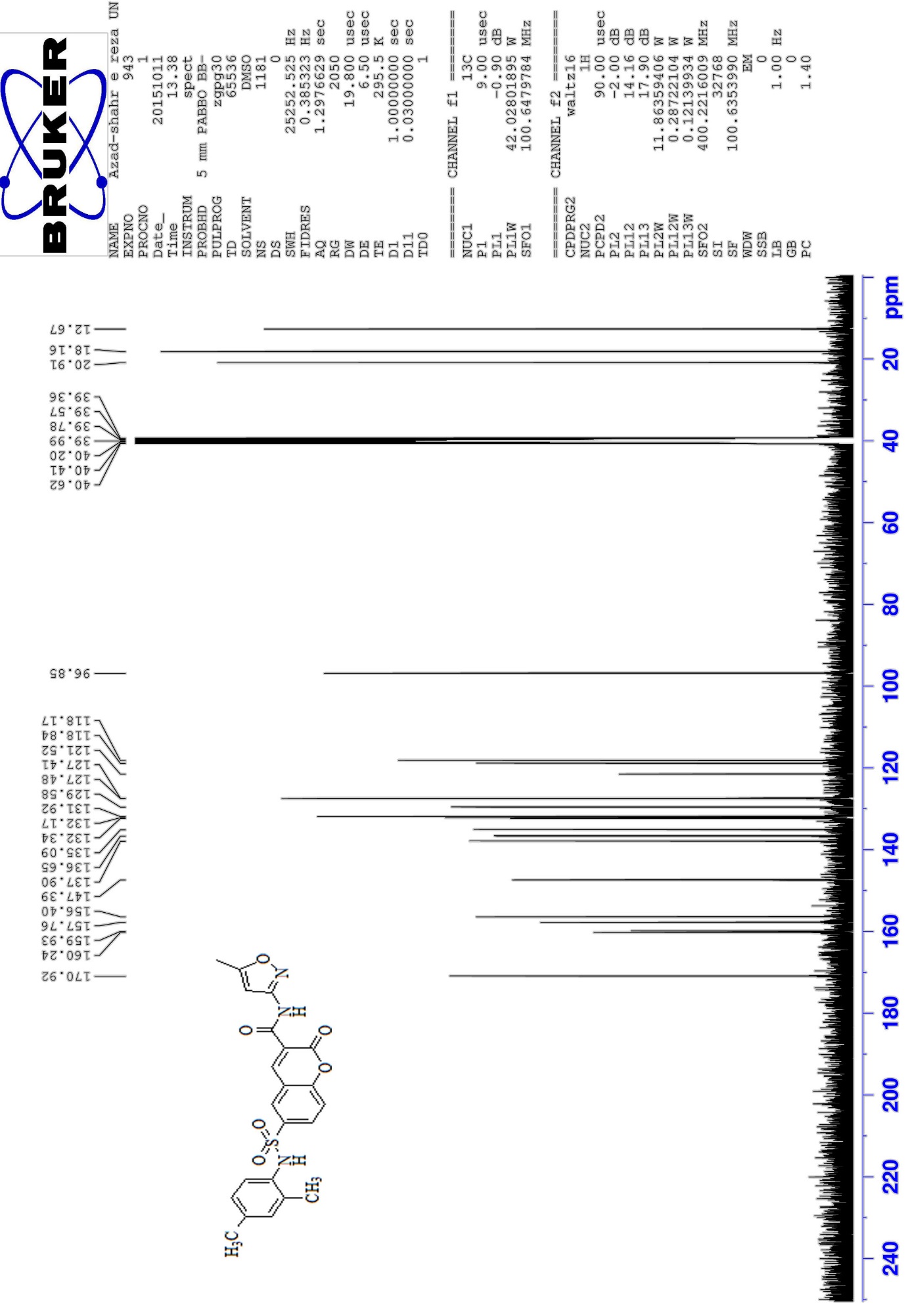 |
| --- | --- |
| **Figure S26. ^1^H NMR spectra of 6-(2,4-Dimethyl-phenylsulfamoyl)-2-oxo-2H-chromene-3-carboxylic acid (5-methyl-isoxazol-3-yl)-amide (*9g*)** | 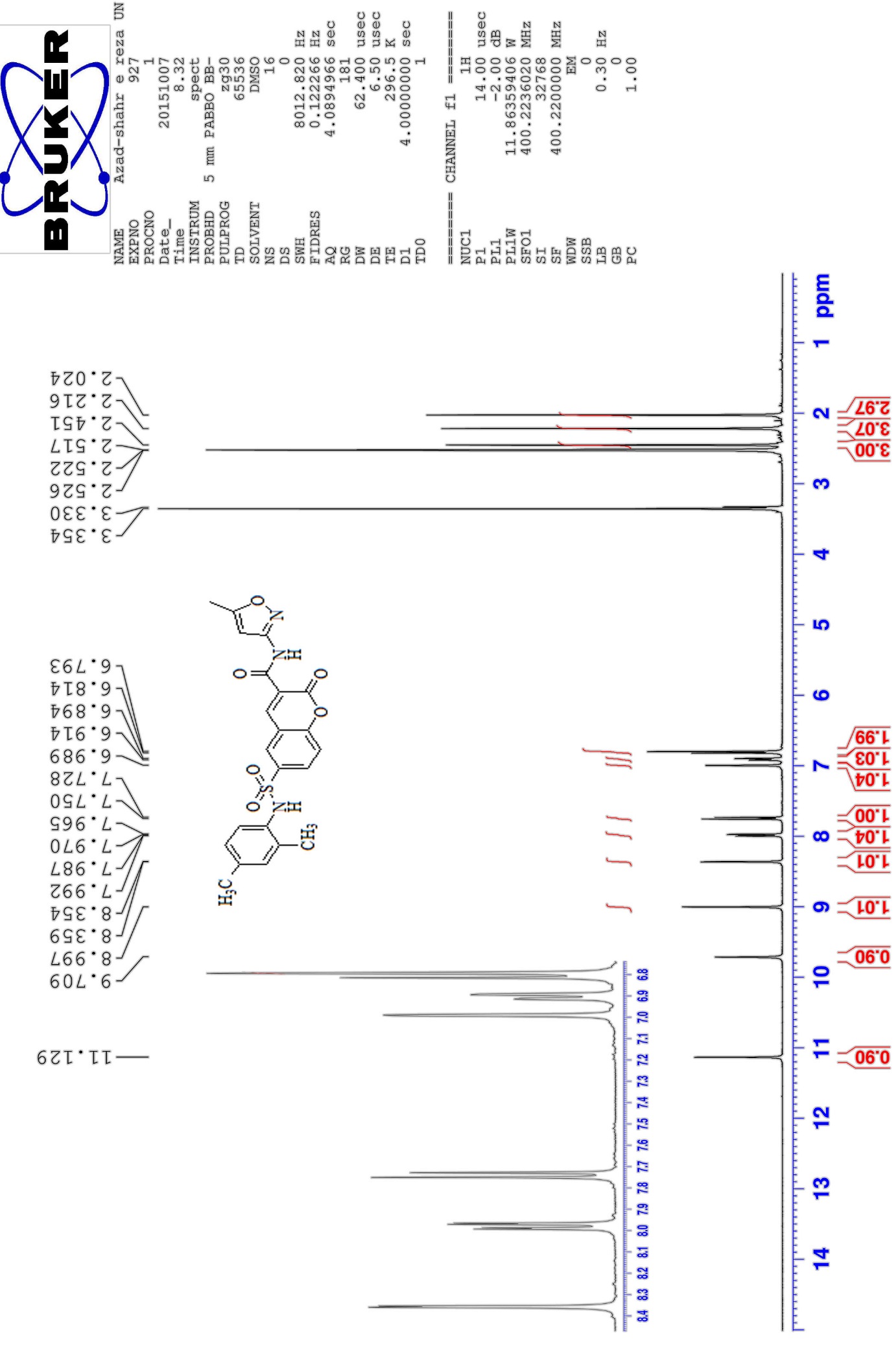 |


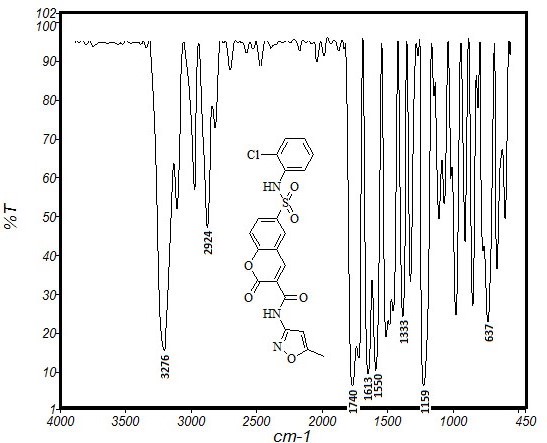


**Figure S27. IR spectra of 6-(2-Chloro-phenylsulfamoyl)-2-oxo-2H-chromene-3-carboxylic acid (5-methyl-isoxazol-3-yl)-amide (*9h*)**

| **Figure S28. ^13^C NMR spectra of 6-(2-Chloro-phenylsulfamoyl)-2-oxo-2H-chromene-3-carboxylic acid (5-methyl-isoxazol-3-yl)-amide (*9h*)** | 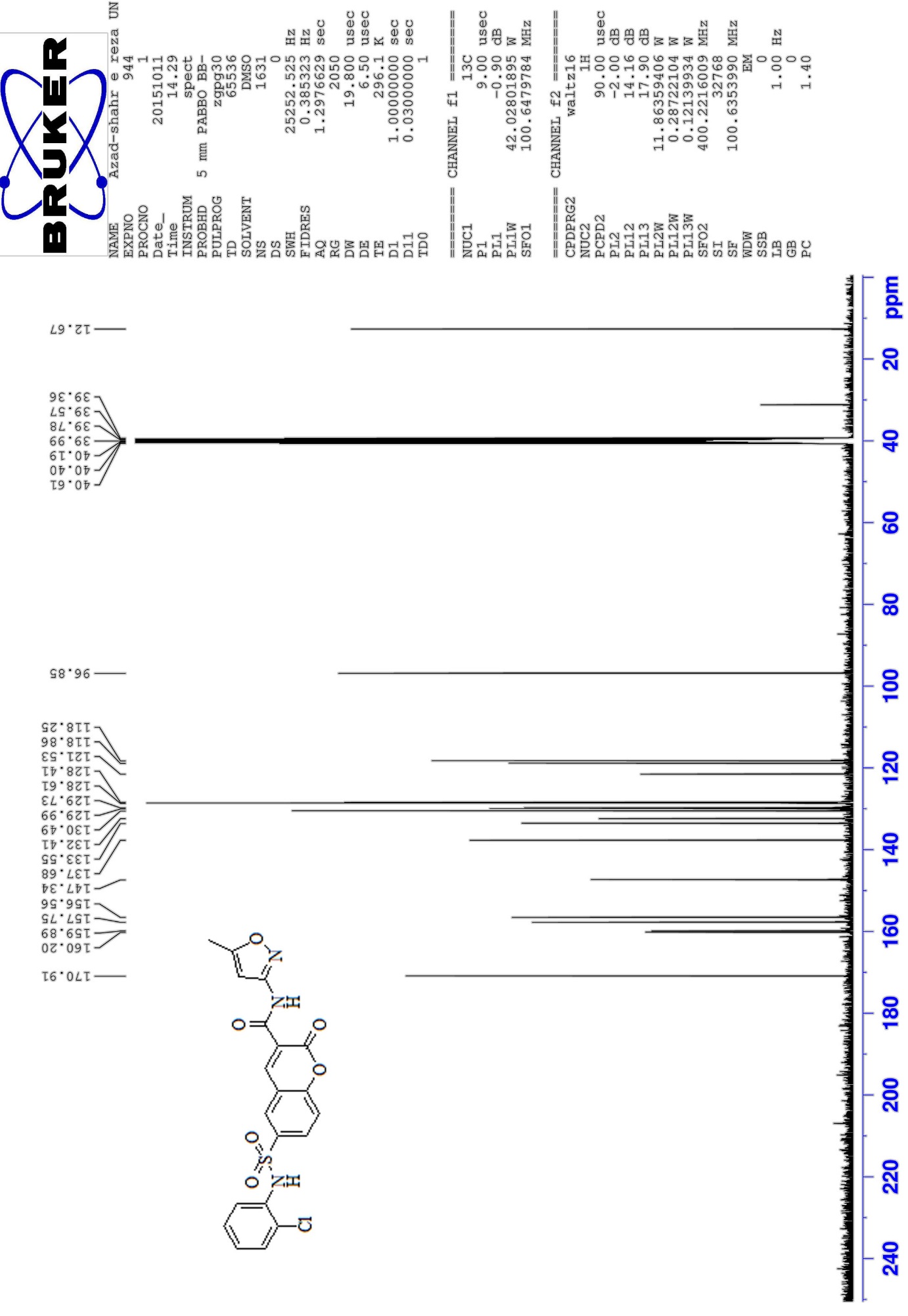 |
| --- | --- |
| **Figure S29. ^1^H NMR spectra of 6-(2-Chloro-phenylsulfamoyl)-2-oxo-2H-chromene-3-carboxylic acid (5-methyl-isoxazol-3-yl)-amide (*9h*)** | 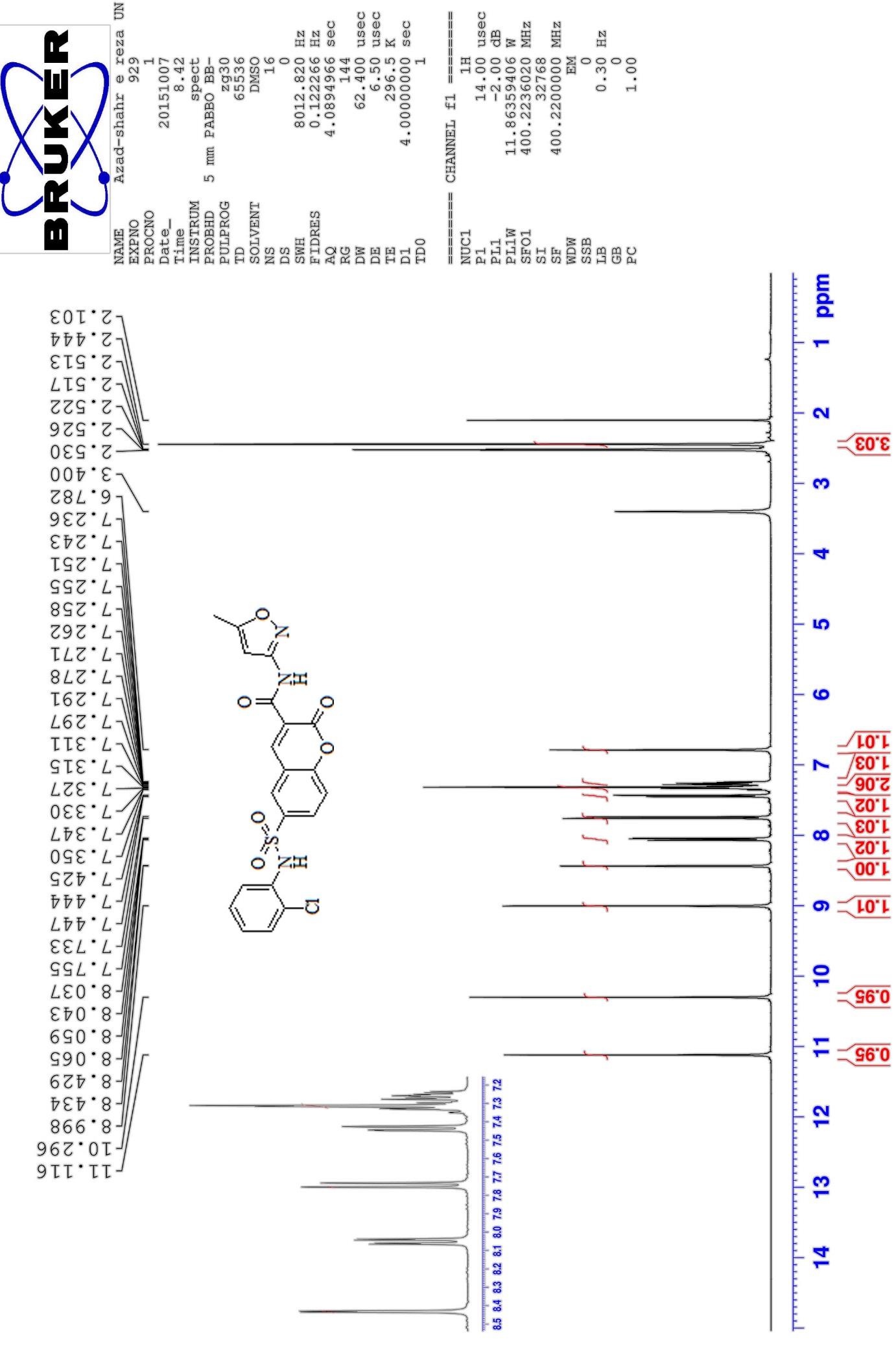 |


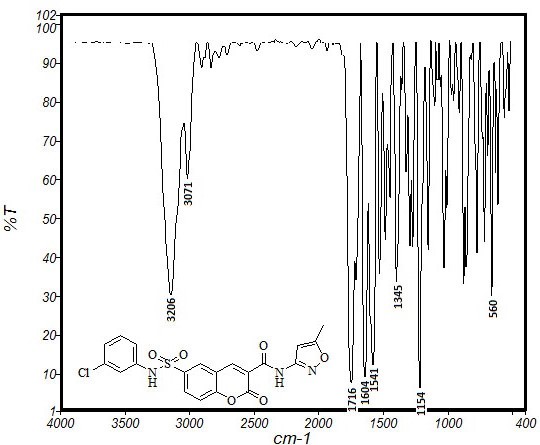


**Figure S30. IR spectra of 6-(3-Chloro-phenylsulfamoyl)-2-oxo-2H-chromene-3-carboxylic acid (5-methyl-isoxazol-3-yl)-amide (*9i*)**

| **Figure S31. ^13^C NMR spectra of 6-(3-Chloro-phenylsulfamoyl)-2-oxo-2H-chromene-3-carboxylic acid (5-methyl-isoxazol-3-yl)-amide (*9i*)** | 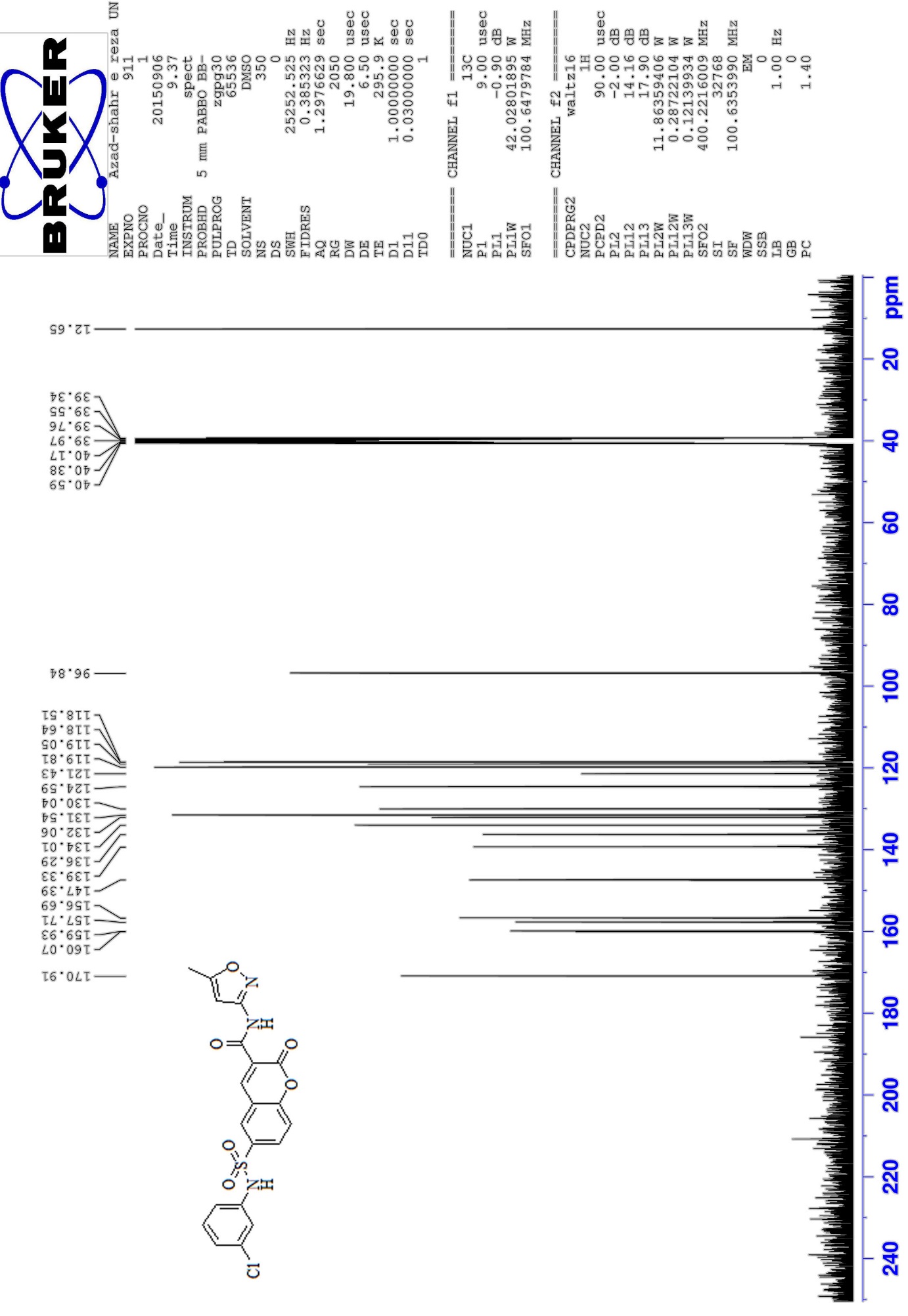 |
| --- | --- |
| **Figure S32. ^1^H NMR spectra of 6-(3-Chloro-phenylsulfamoyl)-2-oxo-2H-chromene-3-carboxylic acid (5-methyl-isoxazol-3-yl)-amide (*9i*)** | 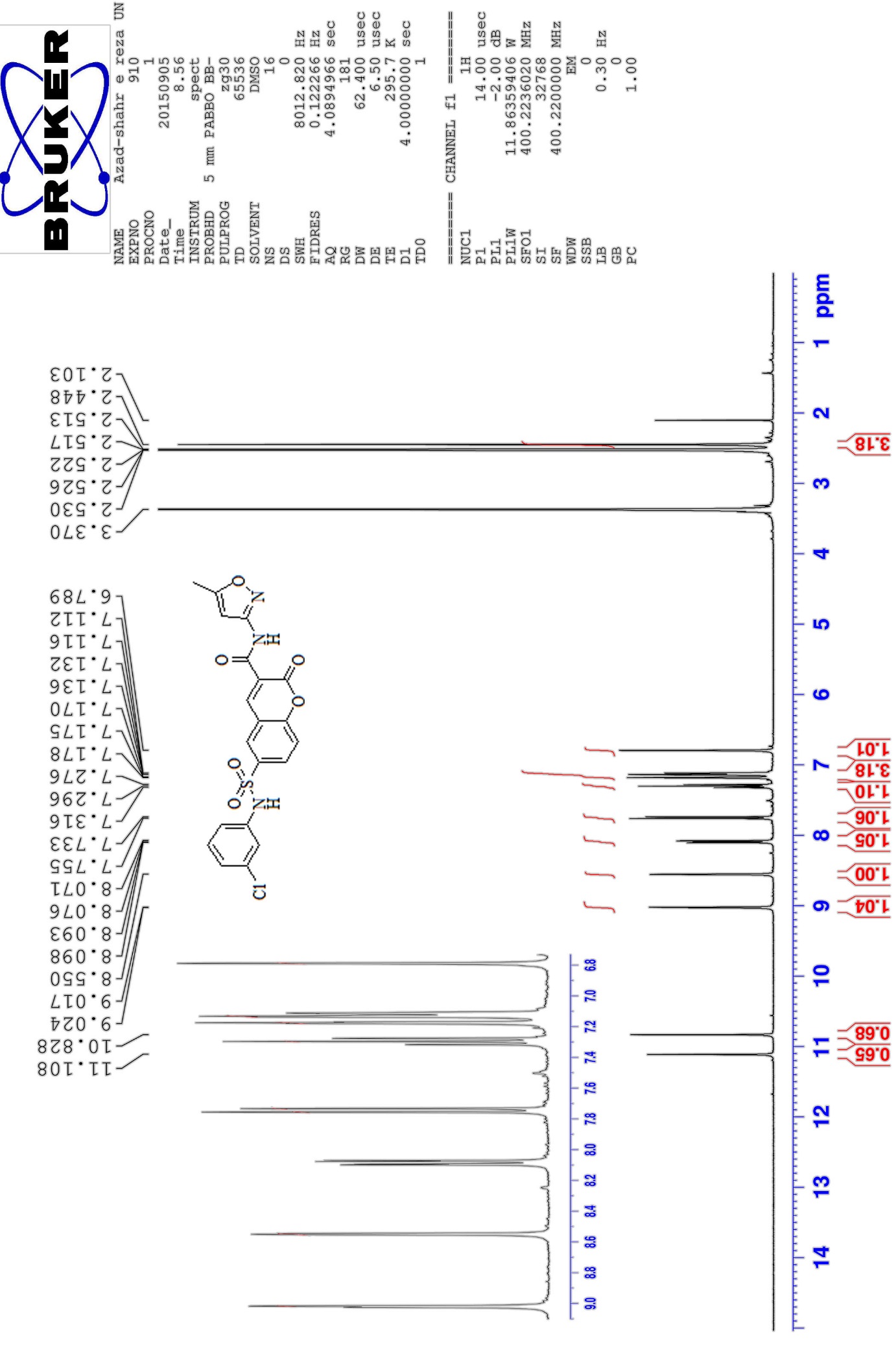 |


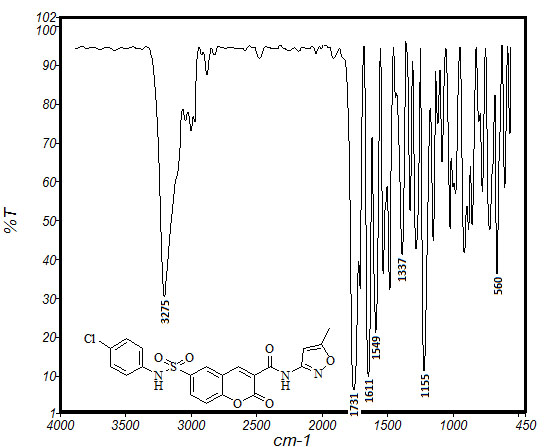


**Figure S33. IR spectra of 6-(4-Chloro-phenylsulfamoyl)-2-oxo-2H-chromene-3-carboxylic acid (5-methyl-isoxazol-3-yl)-amide (*9j*)**

| **Figure S34. ^13^C NMR spectra of 6-(4-Chloro-phenylsulfamoyl)-2-oxo-2H-chromene-3-carboxylic acid (5-methyl-isoxazol-3-yl)-amide (*9j*)** | 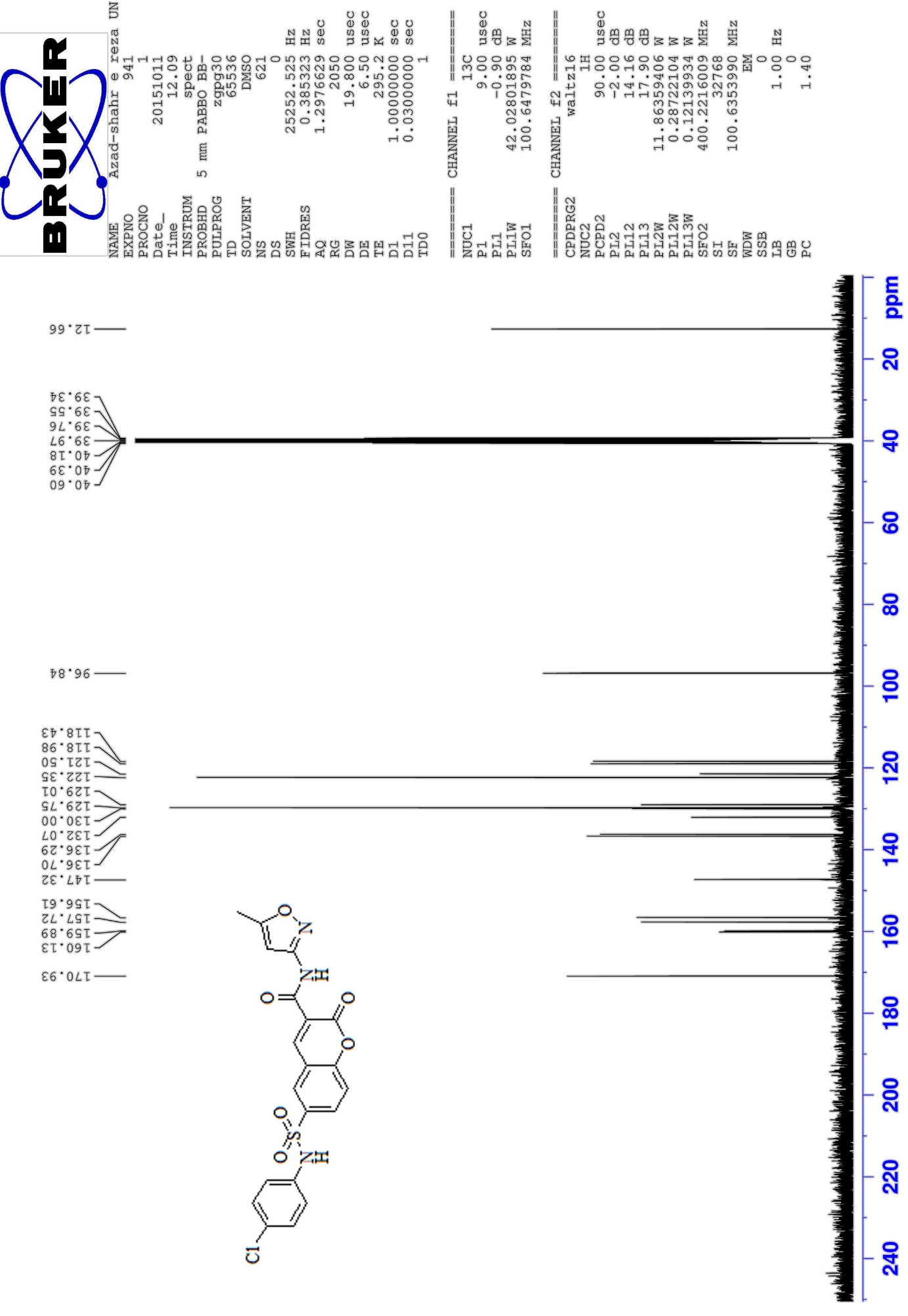 |
| --- | --- |
| **Figure S35. ^1^H NMR spectra of 6-(4-Chloro-phenylsulfamoyl)-2-oxo-2H-chromene-3-carboxylic acid (5-methyl-isoxazol-3-yl)-amide (*9j*)** | 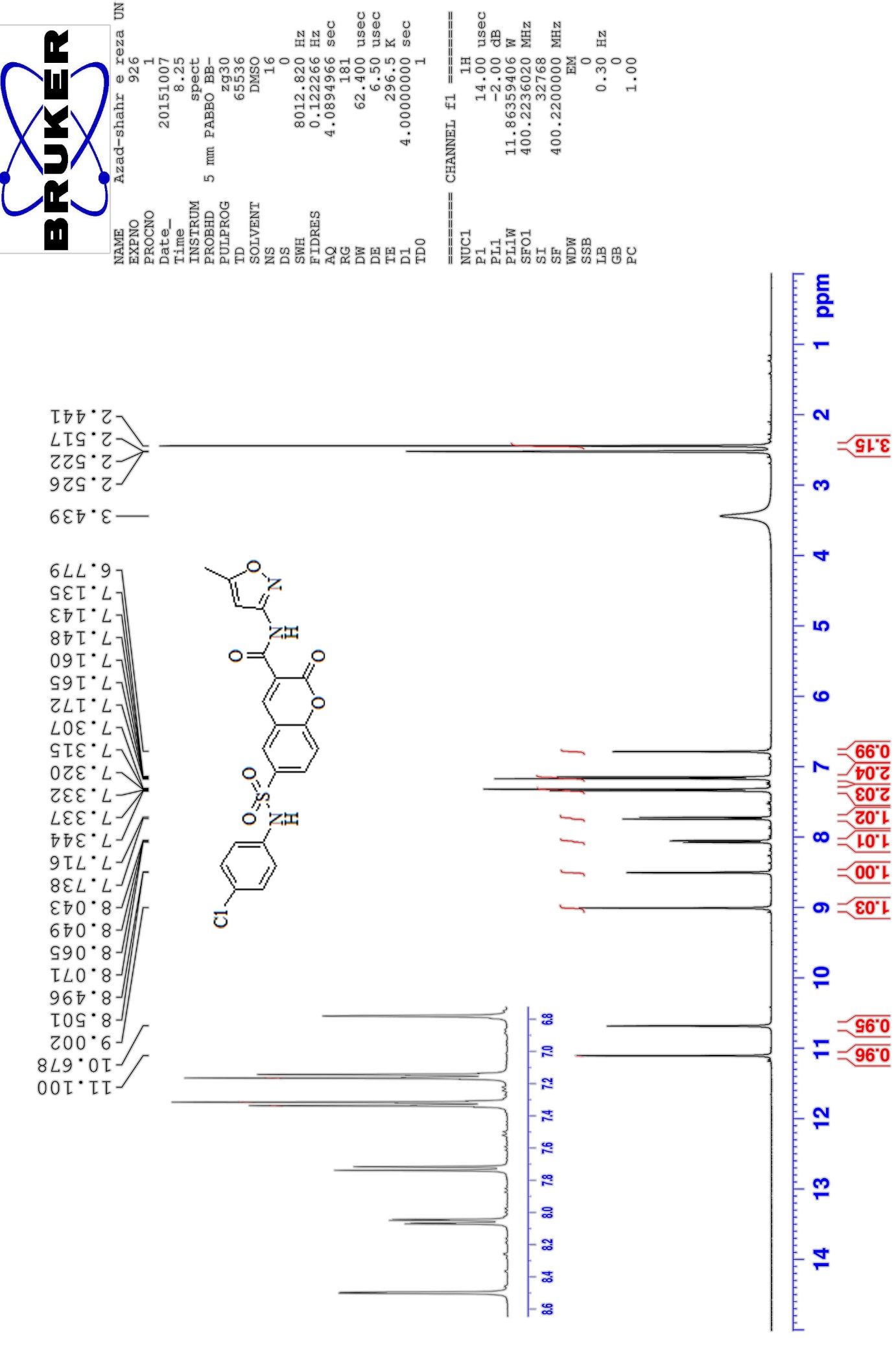 |


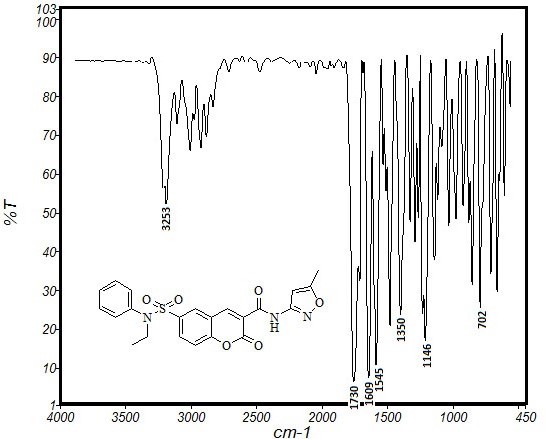


**Figure S36. IR spectra of 6-(Ethyl-phenyl-sulfamoyl)-2-oxo-2H-chromene-3-carboxylic acid (5-methyl-isoxazol-3-yl)-amide (*9k*)**

| **Figure S37. ^13^C NMR spectra of 6-(Ethyl-phenyl-sulfamoyl)-2-oxo-2H-chromene-3-carboxylic acid (5-methyl-isoxazol-3-yl)-amide (*9k*)** | 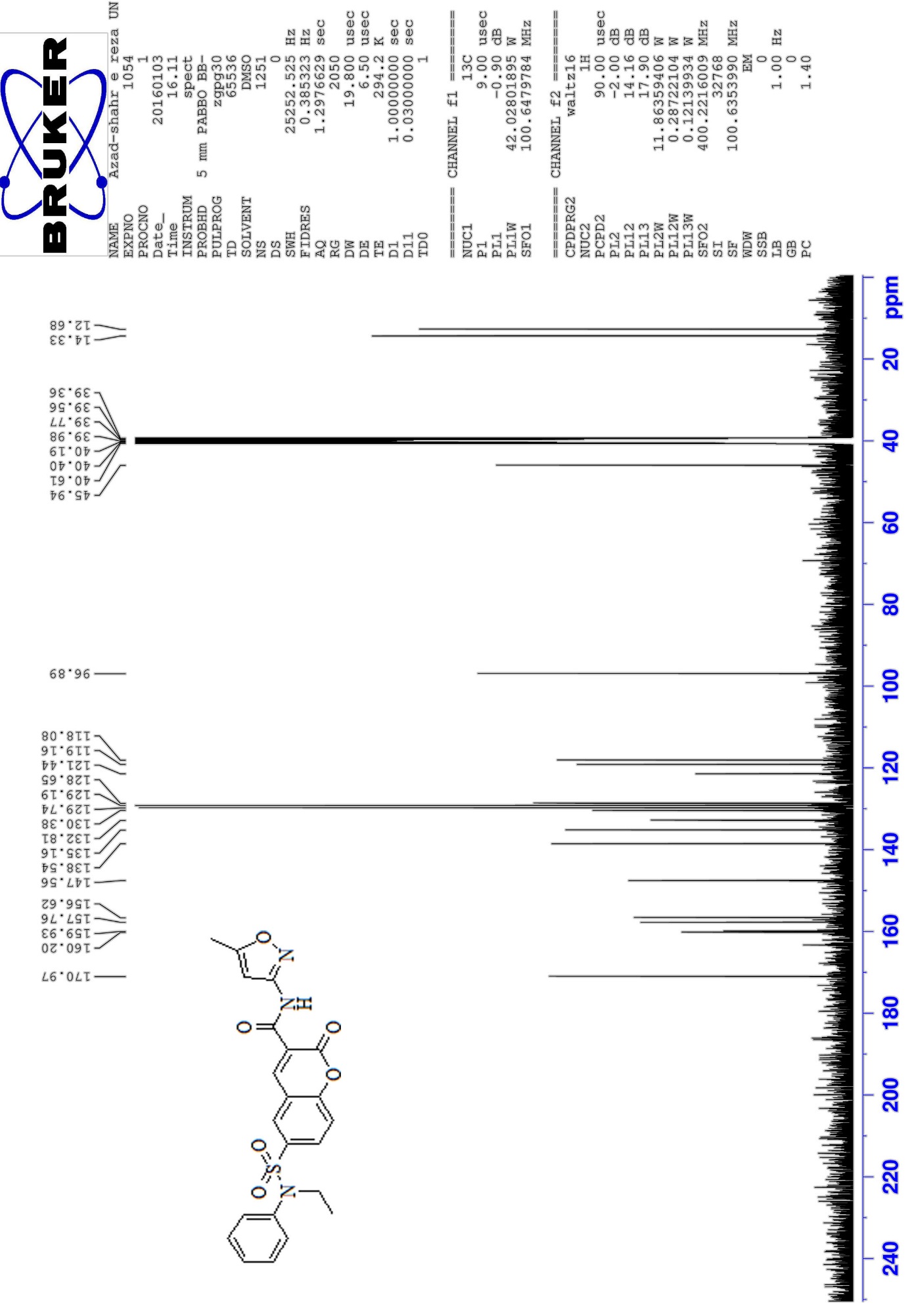 |
| --- | --- |
| **Figure S38. ^1^H NMR spectra of 6-(Ethyl-phenyl-sulfamoyl)-2-oxo-2H-chromene-3-carboxylic acid (5-methyl-isoxazol-3-yl)-amide (*9k*)** | 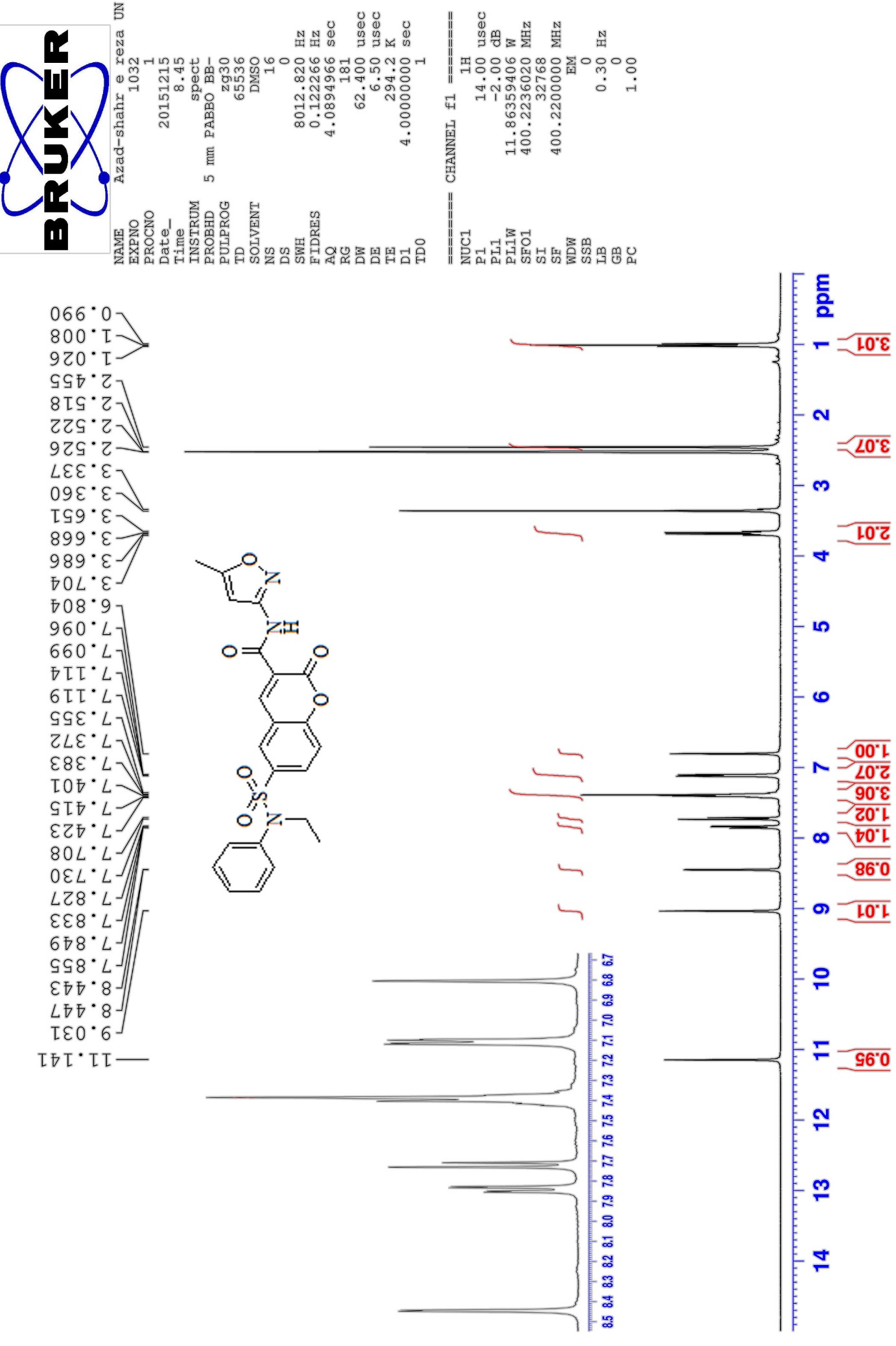 |


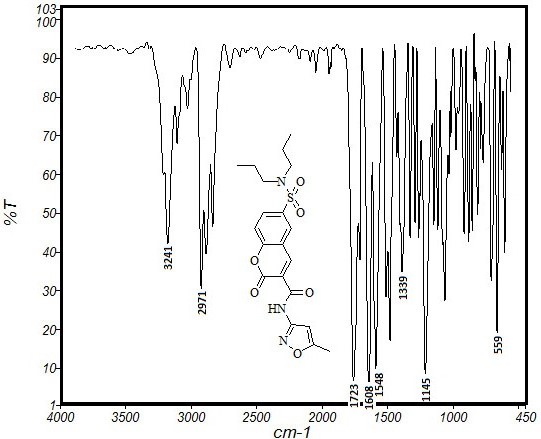


**Figure S39. IR spectra of 6-Dipropylsulfamoyl-2-oxo-2H-chromene-3-carboxylic acid (5-methyl-isoxazol-3-yl)-amide (*9l*)**

| **Figure S40. ^13^C NMR spectra of 6-Dipropylsulfamoyl-2-oxo-2H-chromene-3-carboxylic acid (5-methyl-isoxazol-3-yl)-amide (*9l*)** | 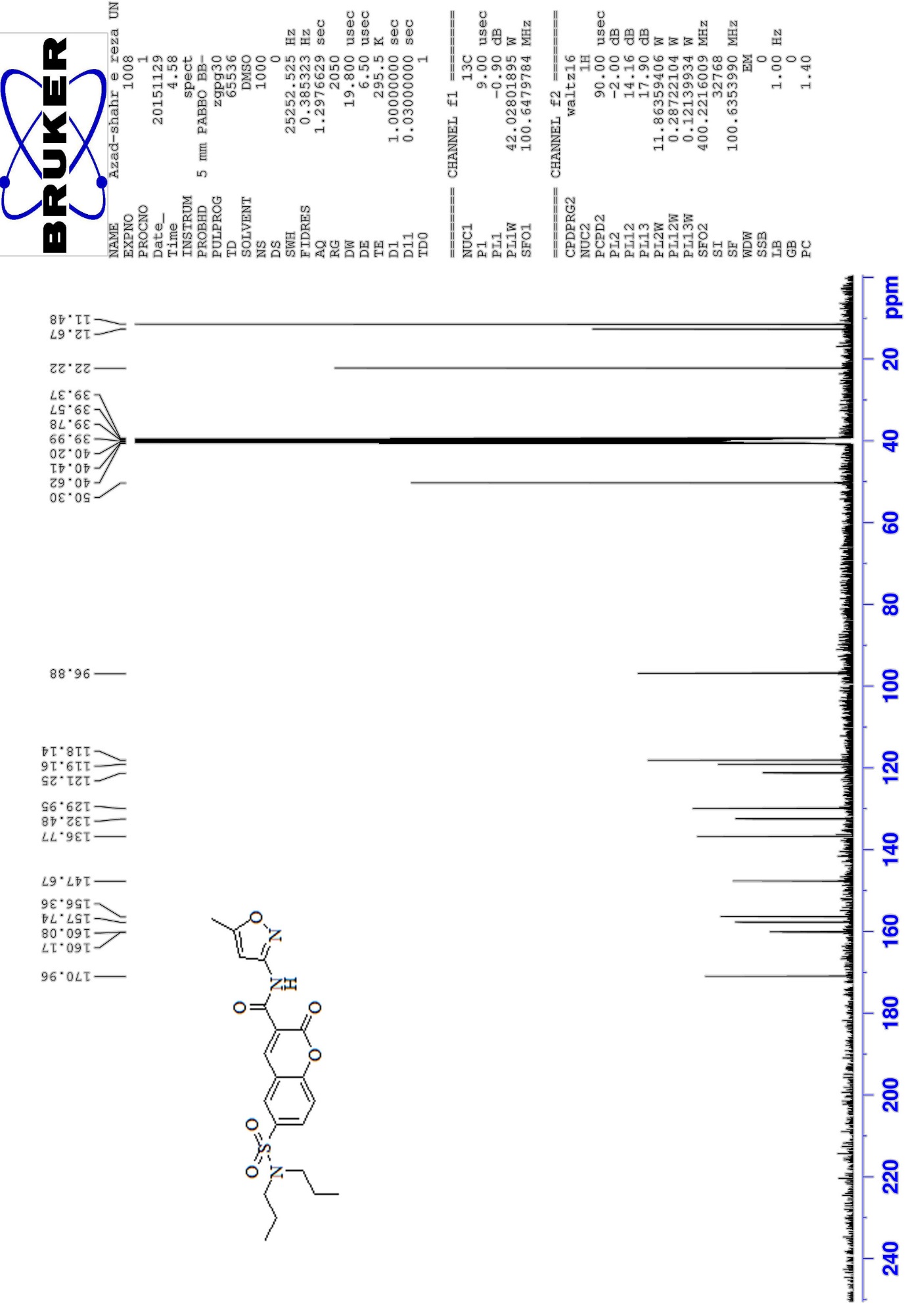 |
| --- | --- |
| **Figure S41. ^1^H NMR spectra of 6-Dipropylsulfamoyl-2-oxo-2H-chromene-3-carboxylic acid (5-methyl-isoxazol-3-yl)-amide (*9l*)** | 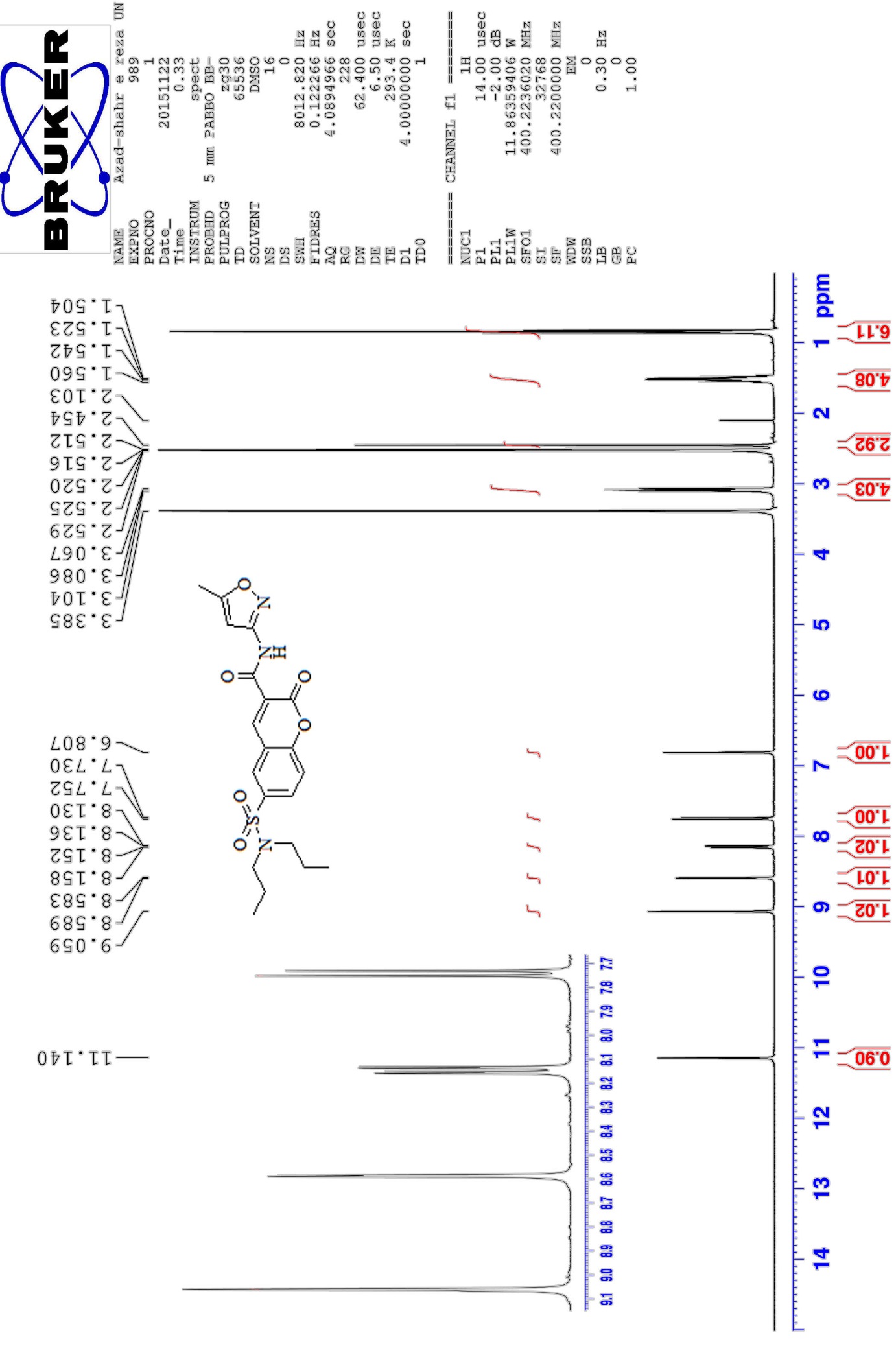 |


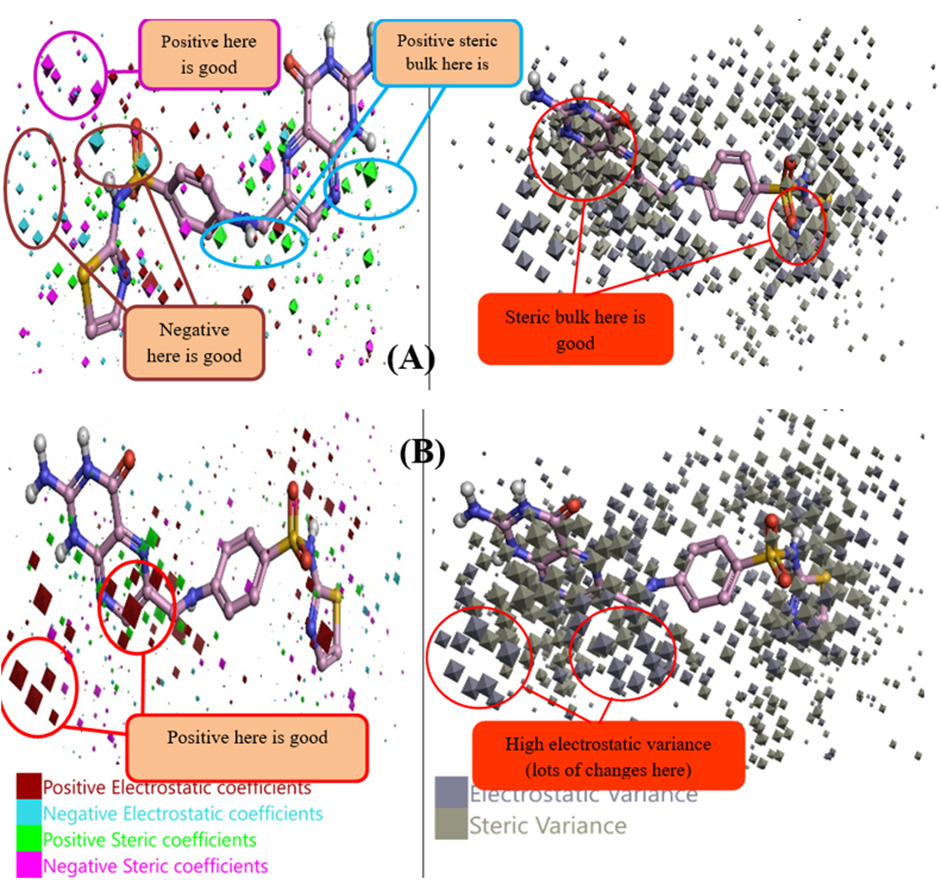


Figure S42. molecular insight of sulfonamide (references molecules) describing the coefficient and variance field points bioactivity via the derived 3D-QSAR model. Model coefficient field points in green color (positive steric coefficient), red color (positive electrostatics) and cyan color (negative electrostatics) present the region with a significant impact on higher activity. High electrostatic variance and high steric variance field points demonstrate the region of high modifications and points with low variance illustrates in that region with less or no change.


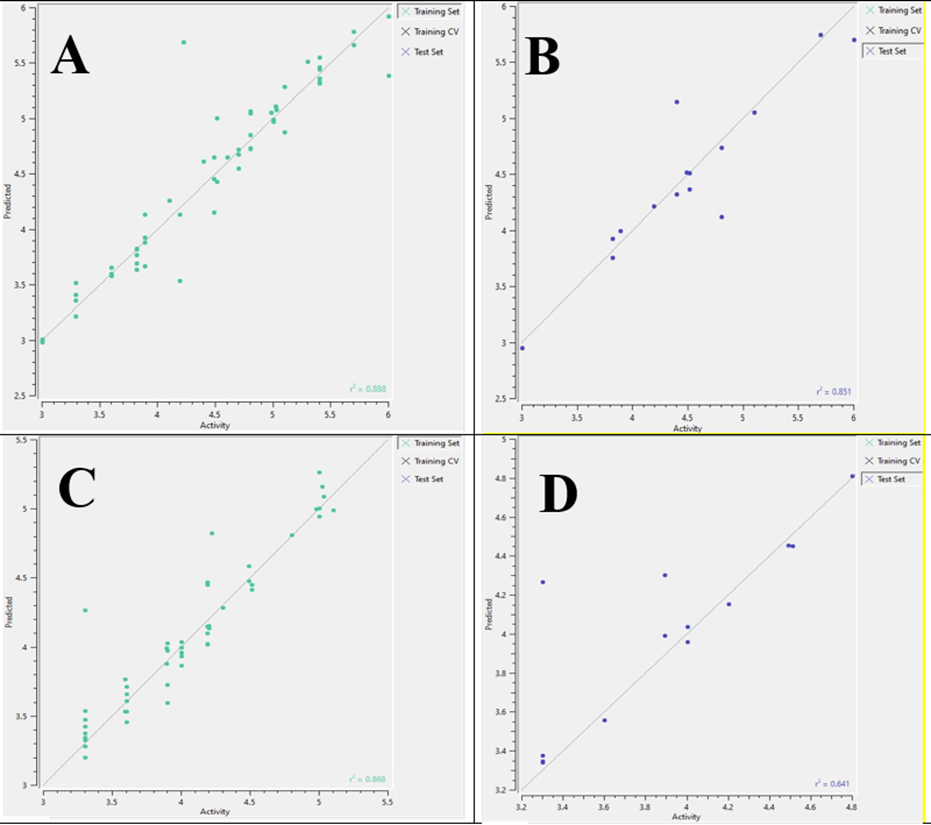


Figure S43. Predicted versus experimental activity plots of the training and test set's molecules. A and B column display the *S. aureus* and C and D *E.* coli, respectively. The $r^{2}$ of training and test sets of all models were beyond 0.6 so all of these data verify that this model is the accurate and applicable to predict MIC of the compounds.
